# Antibacterial impact of biosynthesized zinc oxide nanoparticles on uropathogenic *Escherichia coli* and in vivo assessment of physiological and histological alterations

**DOI:** 10.1038/s41598-025-98060-6

**Published:** 2025-05-05

**Authors:** Magdy A. Abu-Gharbia, Mohamed Farag El-Sayed, Jehan M. Salem, Walaa Magdy Abd-Elsamei, Gehad Al-Arabi

**Affiliations:** 1 Botany and Microbiology Department, Faculty of Science, Sohag University, Sohag, 82524 Egypt; 2 Zoology Department, Faculty of Science, Sohag University, Sohag, 82524 Egypt

**Keywords:** Microbiology, Physiology, Urology

## Abstract

Zinc oxide nanoparticles (ZnO NPs) possess various medical potentials that qualify them to be promising antibacterial agents, particularly for uropathogens. The present study investigated in vitro and in vivo antibacterial impact of biosynthesized ZnO NPs against uropathogenic *E. coli* strain. Values of minimum inhibitory concentration (MIC) and minimum bactericidal concentration (MBC) of ZnO NPs were detected to be 3.2 mg/mL and 3.9 mg/mL, respectively. The in vivo study included twenty-four female albino rats that were divided into four equal groups: group 1 (control), group 2 (infected), group 3 (infected + ZnO NPs), and group 4 (ZnO NPs). The bactericidal efficacy of ZnO NPs (50 mg/Kg) was confirmed by a recovery percentage of 83.3% after the fourth dose and a survival rate of 100% after eight doses. Erythrocytosis and thrombocytopenia were observed in the infected group, while ZnO NPs-administrated groups exhibited normal red blood cells and platelets counts, and a significant increase in white blood cells count. A significant decrease in urea level and a slight increase in liver enzymes were observed in the infected group, unlike ZnO NPs-administrated groups. Moreover, ZnO NPs-administrated groups exhibited a significant decrease in uric acid and glucose levels. The histological sections of vital body organs showed the aggressive bacterial-induced inflammatory response in stomach, liver, spleen, kidney, and heart of the infected group, whereas ZnO NPs-treated group exhibited effective suppression of the bacterial infection.

## Introduction

Urinary tract infection (UTI) is considered a serious public health problem with substantial economic and medical burdens^1^. Among several different uropathogens, bacteria, particularly *Escherichia coli*, are the major causative agents of UTI^2^. Due to the misuse of antibiotics, uropathogens have developed resistance to many antimicrobial agents, leading to a therapeutic challenge^3^. The prevalence of drug-resistant bacteria has provoked the need to develop novel drug molecules for effective treatment of infections. Nanotechnology has emerged as a promising therapeutic solution for this dilemma^4^. Nanoparticles (NPs) range mainly from 1 to 100 nm in size. The nanoscale dimension provides NPs with a large surface area-to-volume ratio and thus highly specific properties, including optical, electronic, and medicinal properties^5^. Among various metal and metal oxide nanoparticles reported to have biological properties, zinc oxide nanoparticles (ZnO NPs) are commonly considered^6^.

Zinc attracts a little attention during the assessment of NPs toxicity as it is an essential trace element in the human body and is commonly added as a nutritional supplement. Nanosized ZnO particles have garnered interest due to their unique properties, distinct from bulk-sized ZnO particles. When the particle size of ZnO decreases, its ultraviolet filtering efficiency, chemical reactivity, transparency, and dispersion properties increase, adding attractive characteristics for commercial application^7^. Nanosized ZnO possesses various biological activities, including antibacterial, antifungal^8^, antidiabetic, anticancer^9^, anti-inflammatory, wound healing, antioxidant^10^, and optical properties^5^. In addition, ZnO NPs are inexpensive to produce, biosafe and highly biocompatible. Their fast electron transport kinetics rate makes them convince for use in the biological applications^11^. Many studies have illustrated the antimicrobial activity of ZnO NPs against a broad spectrum of pathogenic bacteria^12–14^.

Chemical and physical synthesis of NPs can release toxic byproducts that are extremely hazardous to the environment. These byproducts can cause health-related issues^15^. To address this concern, the biosynthesis of NPs was adopted to reduce the toxicity of metals during the bioreduction process^16^. Plant-mediated NPs are coated with a variety of phytochemicals, which may enhance their antibacterial activity^17^. These pharmacologically active biomolecules, which are mainly known to have a significant therapeutic effect against a wide range of human pathogens, allow multiple ligand-based conjugation of NP with receptors on microbial membranes. These phytochemicals contribute to the antibacterial activity of NPs by initiating a cascade of events, such as reactive oxygen species (ROS) generation, enzyme inhibition, disruption of biofilm, protein denaturation, cell membrane integrity disruption, and/or accelerating the process^18^.

NPs can function as antibacterial agents through adopting a series of mechanisms. Loss of cell membrane integrity was reported to be the major cause of bactericidal effect of ZnO NPs on *E. coli* cells^19^. The oxidative stress induced by generation of ROS is another important mechanism. Persistent oxidative damage leads to DNA, protein, and lipid damage. DNA fragmentation, such as single- and double-stranded breakage, adduct formation, and DNA cross-linkage with proteins, are common ways of DNA damage caused by exposure to NPs. Cell failure to repair damaged DNA may lead to cell apoptosis or necrosis^20^.

Although ZnO NPs are metabolizable and are generally recognized as safe, the tolerance of living tissues to absorbed ZnO NPs should be carefully evaluated. Many researchers have tried to assess the hematological and histological effects of NPs through animal models^21–24^. Generally, ZnO NPs can be internalized as either Zn^2+^ free ions or NPs by cells. The circulatory system is the first area of the body where ZnO NPs can reach after exposure in any manner. The liver, kidneys, and spleen are the primary targets of ZnO NPs and where high concentrations of ZnO NPs are found^25^. Assorted sizes and particle shapes of ZnO NPs may impact their cytotoxicity^26^. Several in vitro and in vivo studies have reported that ZnO NPs are hazardous regarding their size and dose^27–30^. The NP size is correlated with important properties, such as surface properties, solubility, and chemical reactivity, which have critical effects on the interactions between nanomaterials and biomolecules, and subsequently on in vivo nanotoxicological behavior. A reduction in size could increase the specific surface area of NPs, promoting their accumulation and enhancing interactions with biomolecules^31^. However, another study conducted to assess the acute oral toxicity of ZnO NPs indicated that both 20-nm and 120-nm ZnO particles are similar to the normal ZnO compound and relatively nontoxic to all tested mice, according to the Globally Harmonized System (GHS) classification criteria^32^.

The future perspective of plant-mediated ZnO NPs highlights the need for more extensive experiments on living tissues to extend the laboratory-based work to the industrial scale and real treatment. With this insight, the present study aims to investigate the in vitro and in vivo antibacterial effect of biosynthesized ZnO NPs, considering hematological, biochemical, and histological changes on a rat model.

## Results

### Bacterial strain and culture conditions

**Fig. 1:**
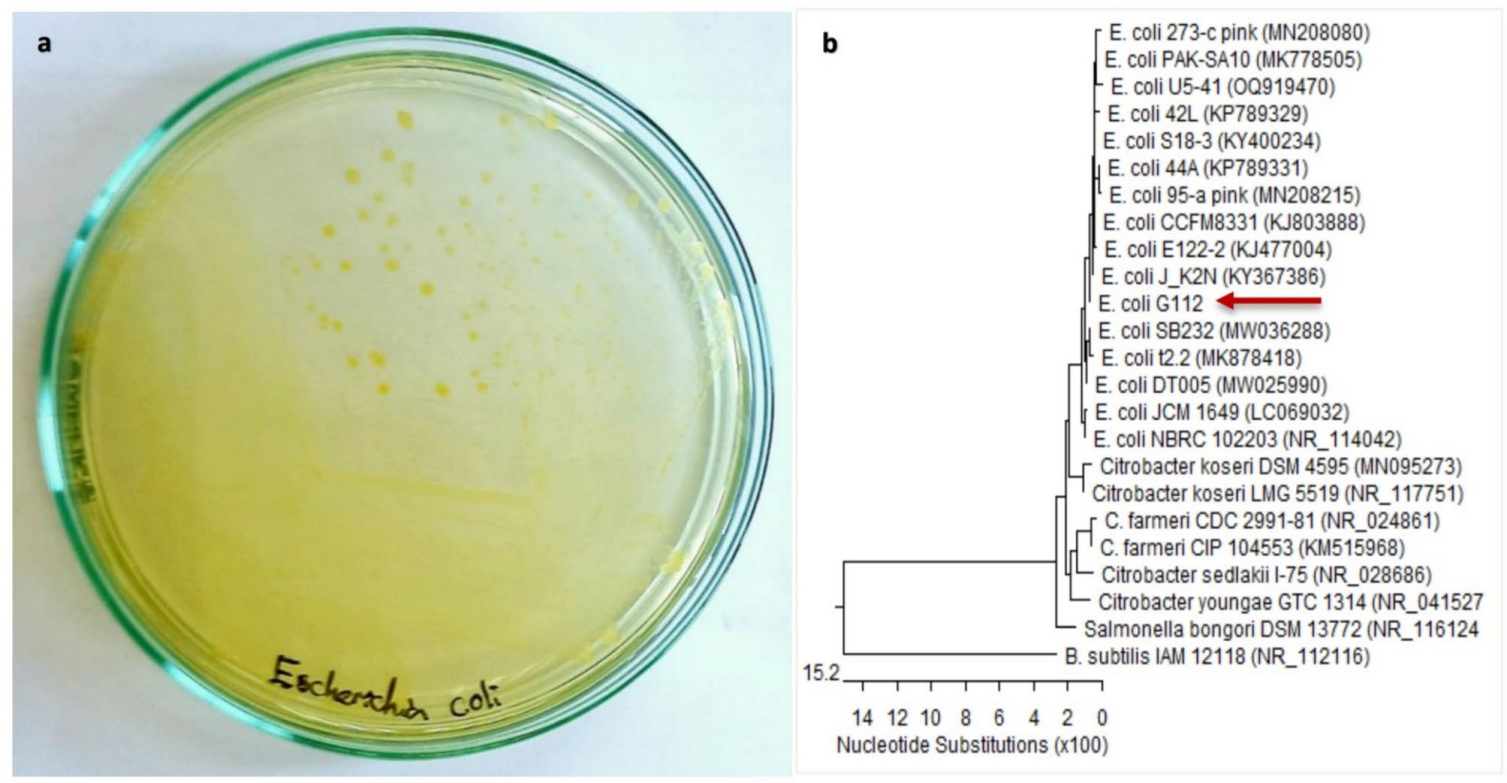
Identification of the bacterial strain. (**a**) *Escherichia coli* strain appeared on CLED agar as lactose fermenter, opaque, bright yellow colonies “reproduced from ref 2”. (**b**) Phylogenetic tree based on 16S rRNA sequence of *Escherichia coli* (arrowed) aligned with closely related strains accessed from the GenBank. *Bacillus subtilis* is included in the tree as outgroup strain. *Escherichia coli* OR064346 showed 99.72–99.93% identity and 99–100% coverage with several strains of the same species including the type strain *E. coli* NBRC102203 (NR_114042).

The bacterial strain appeared on CLED agar as lactose fermenter, opaque, bright yellow colonies with slightly deeper center^2^ (Fig. 1a). Microscopic examination showed short rods Gram-negative bacteria. Phenotypic identification confirmed that the bacterial strain was ESBL-producing *E. coli*. Molecular identification of the bacterial strain was confirmed to be *Escherichia coli* OR064346. Phylogenetic analysis of sequences revealed (99.72–99.93%) identity and (99–100%) coverage with several strains of the same species (Fig. 1b).

### Biosynthesis and characterization of ZnO NPs

**Fig. 2:**
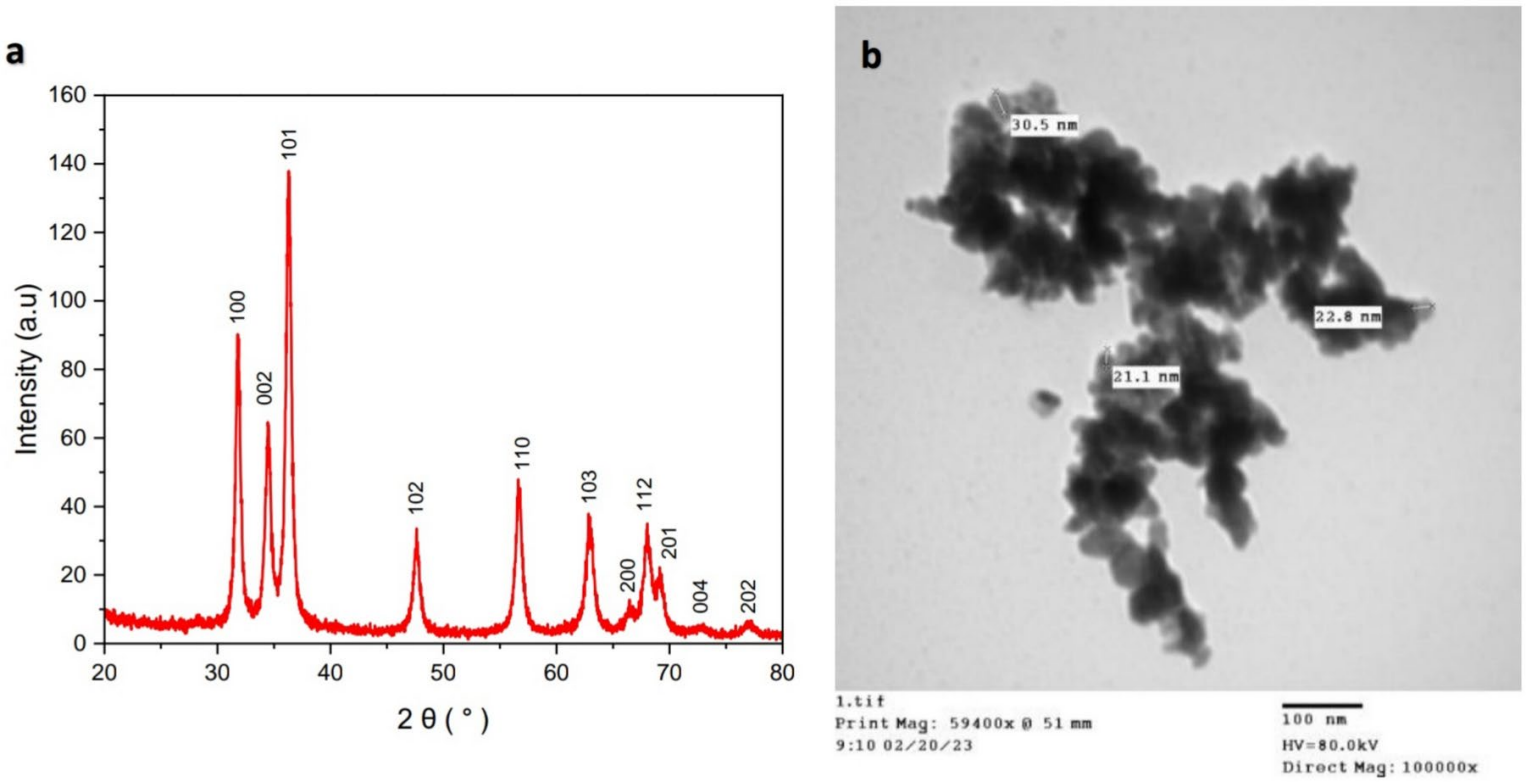
Characterization of biosynthesized ZnO NPs. (**a**) XRD patterns confirmed the highly crystalline hexagonal wurtzite structure of ZnO NPs. (**b**) TEM image exhibited the hexagonal shape of ZnO NPs. “reproduced from ref^33^”.

The biosynthesis of ZnO NPs was performed via a remarkably simple approach. The bioreduction of Zn^2+^ ion by *Aloe vera* leaves extract was detected using UV–Vis spectrophotometer at a wavelength of 383 nm with an absorbance value of 5.71^33^. X-ray diffraction (XRD) analysis provided information about the crystalline structure of the biosynthesized ZnO NPs^33^ (Fig. 2a). The XRD pattern confirmed the highly crystalline hexagonal wurtzite structure of ZnO NPs with lattice parameters of a = 3.24 Å, b = 3.23 Å, and c = 5.18 Å which matched JCPDS card no. 36-1451. The average crystallite size of ZnO NPs was calculated at the most intense peaks using Scherrer’s equation: D = 0.98 λ/β cos θ, where the wavelength of X-ray “λ” = 1.54060, β is full width at half maximum in radians, and θ diffraction angle. The average mean crystallite size was calculated to be 16.7 nm. Transmission electron microscopy (TEM) exhibited that the majority of ZnO NPs was hexagonal-shaped with a median particle size of ~ 20 nm^33^ (Fig. 2b).

### Testing in vitro antibacterial impact of ZnO NPs

**Fig. 3:**
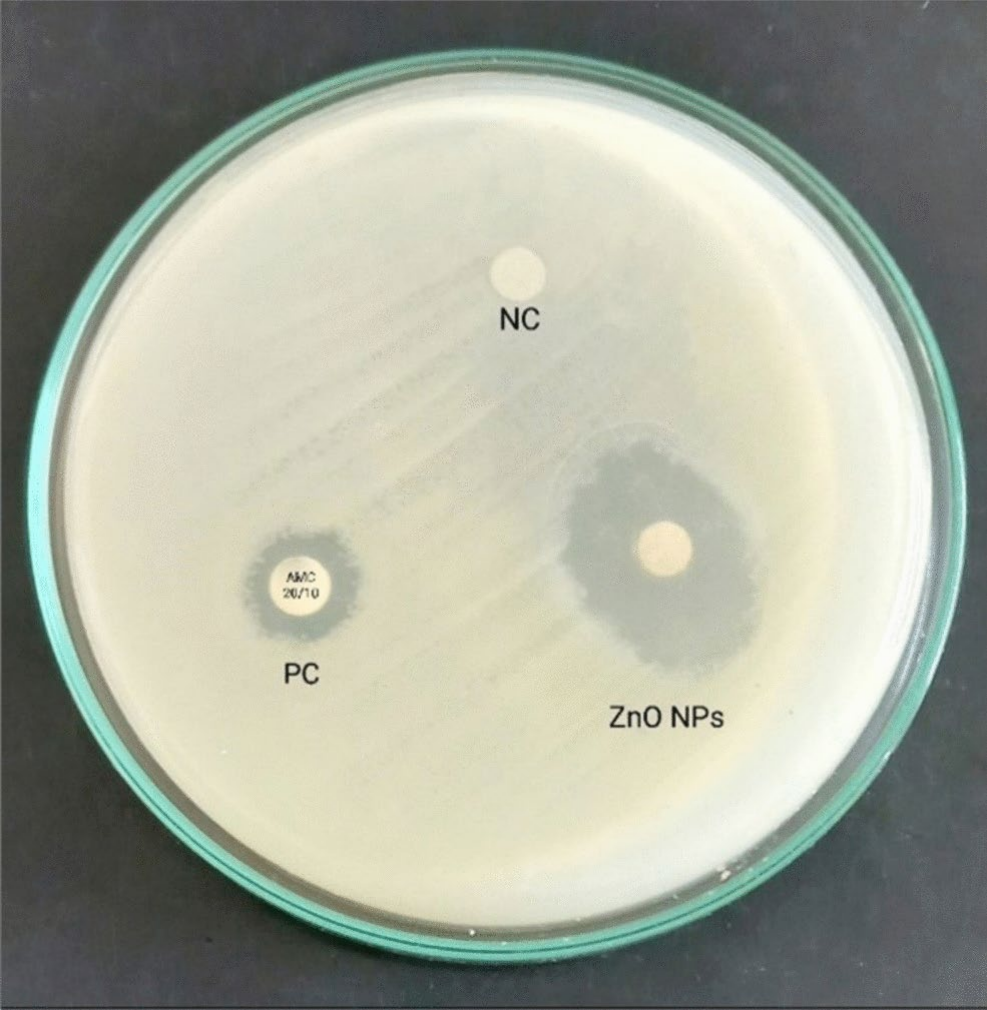
Antibacterial effect of ZnO NPs on *E. coli* compared to amoxicillin/clavulanate (20/10 μg) disc as a positive control “PC” and glycerol and water (4:1 v/v) disc as a negative control “NC”.

The in vitro antibacterial impact of ZnO NPs was confirmed using disk diffusion susceptibility test (Fig. 3). The diameters of inhibition zones confirmed the efficacy of ZnO NPs against *E. coli* strain compared to amoxicillin/clavulanate (Table 1). The MIC was detected using 96-well microtiter plate assay as the first concentration of ZnO NPs that inhibited the visible bacterial growth, and then the MBC was detected as the concentration that destroyed all the bacterial cells. Values of MIC and MBC were detected to be 3.2 mg/mL and 3.9 mg/mL, respectively.

**Table 1:** In vitro antibacterial impact of ZnO NPs on *E. coli* strain.

Bacterial strain	Zone of inhibition (mm)			MIC (mg/mL) of ZnO NPs	MBC (mg/mL) of ZnO NPs
	ZnO NPs (5 mg/mL)	AMC (20/10 μg)	Glycerol + water (4:1 v/v)		
*E. coli* OR064346	23.33 ± 0.58	9 ± 1	0	3.2	3.9

### Testing in vivo antibacterial impact of ZnO NPs on rat model

#### Animals and experimental groups

**Fig. 4:**
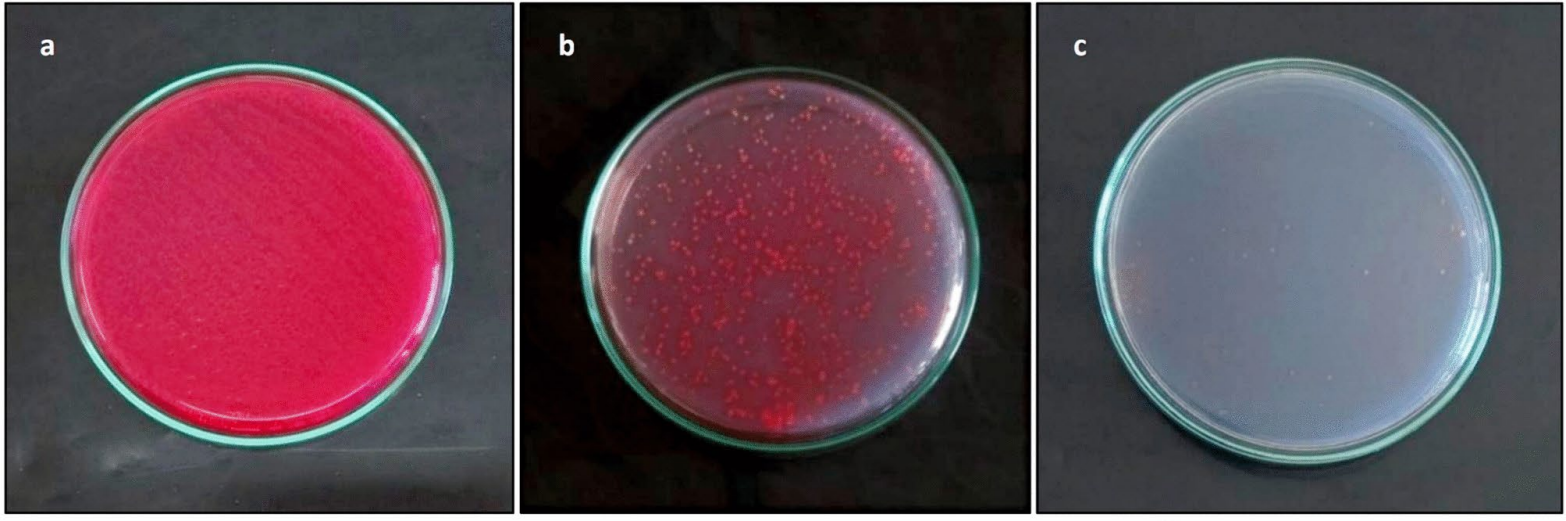
Urine cultures performed on CLED agar with Andrade indicator confirmed the antibacterial effect of ZnO NPs. (**a**) Luxurious growth after 24 h of infection induction. (**b**) Reduction in the bacterial growth after 24 h of applying the 1st dose of ZnO NPs. (**c**) No growth after 24 h of applying the 4th dose of ZnO NPs.

Urine cultures confirmed the in vivo antibacterial impact of ZnO NPs. After 24 h of infection induction in both (infected) and (infected + ZnO NPs) groups, their urine cultures exhibited luxurious growth with uncountable colonies of *E. coli* strain (Fig. 4a). After 24 h of applying the first dose of ZnO NPs, urine cultures of (infected + ZnO NPs) group exhibited a significant reduction in *E. coli* growth (Fig. 4b). After 24 h of applying the fourth dose of ZnO NPs, urine cultures of (infected + ZnO NPs) group exhibited no growth (sterile) in 5 from 6 rats (Fig. 4c). Oral administration of ZnO NPs continued to day 8, however the results were the same, and the recovery percentage was still 83.3%. Cultures of (infected) group exhibited significant growth until day 8, while cultures of (control) and (ZnO NPs) groups remained sterile. No mortality, severe toxicity sign (i.e., diarrhea/hair loss) or abnormal behavior was detected in any of the tested groups until the end of the experiment.

### Blood samples collection

#### Hematological parameters

A significant increase was observed in RBCs count, HGB, and HCT, indicating an erythrocytosis in (infected) group compared to (control) group. No differences were observed in MCV, MCH, MCHC, and RDW-CV in any of the tested groups. Both (infected + ZnO NPs) and (ZnO NPs) groups exhibited a significant increase in WBCs count. No significant difference was observed in neutrophils count in any of the tested groups, while (ZnO NPs) group exhibited a significant increase in lymphocytes count; A lymphocytosis, compared to (control) group. In addition, both of (infected + ZnO NPs) and (ZnO NPs) groups exhibited a significant increase in monocytes count; A monocytosis, compared to (control) group. A significant decrease was observed in PLT count and PCT value, indicating a thrombocytopenia in (infected) group, while (infected + ZnO NPs) and (ZnO NPs) groups showed a significant increase in PLT and PCT compared to (infected) group. No significant differences were observed in MPV or PDW in any of the tested group (Table 2).

**Table 2:** The impact of *E. coli* infection, *E. coli* infection treated with ZnO NPs, and ZnO NPs on the hematological parameters in female albino rats (*Rattus rattus*).

Tested parameter	Group 1 (control)	Group 2 (infected)		Group 3 (infected + ZnO NPs)			Group 4 (ZnO NPs)			
	Mean ± SD	Mean ± SD	P1	Mean ± SD	P1	P2	Mean ± SD	P1	P2	P3
RBC (× 10^6^/μl)	5.16 ± 0.40	5.82 ± 0.30^a^	0.02044	5.44 ± 0.37	0.52691	0.28246	5.67 ± 0.32	0.09028	0.88721	0.67975
HGB (g/dL)	12.75 ± 0.47	13.60 ± 0.42^a^	0.02636	13.12 ± 0.49	0.54891	0.31820	13.26 ± 0.49	0.26482	0.62331	0.94600
HCT (%)	34.1 ± 1.61	37.47 ± 0.80^a^	0.01490	34.98 ± 2.46	0.81329	0.09326	36.45 ± 1.63	0.11987	0.74153	0.47466
MCV (fL)	66.50 ± 2.06	64.56 ± 2.65	0.57613	64.31 ± 2.44	0.46793	0.99774	65.03 ± 2.45	0.77674	0.98580	0.95190
MCH (Pg)	24.80 ± 1.16	23.45 ± 1.23	0.29090	24.18 ± 1.24	0.83765	0.75569	23.95 ± 1.45	0.66413	0.90485	0.98879
MCHC (g/dl)	37.46 ± 0.69	36.26 ± 1.26	0.58236	37.60 ± 1.94	0.99893	0.49733	36.86 ± 2.14	0.91686	0.91686	0.86017
RDW-CV (%)	12.18 ± 0.18	12.06 ± 0.46	0.94169	12.03 ± 0.38	0.88595	0.99845	11.86 ± 0.34	0.43903	0.76954	0.85119
WBCs (× 10^3^/μl)	5.45 ± 0.87	7.38 ± 1.50	0.37555	9.07 ± 2.36^a^	0.02683	0.48583	10.37 ± 2.79^a^	0.00227	0.08136	0.68935
Neutrophils (× 10^3^/μl)	0.344 ± 0.18	0.632 ± 0.42	0.86439	0.802 ± 0.83	0.61309	0.96716	0.105 ± 0.86	0.25765	0.67656	0.90705
Lymphocytes (× 10^3^/μl)	4.58 ± 1.05	6.04 ± 1.72	0.60665	7.38 ± 2.54	0.11083	0.66517	8.34 ± 2.44^a^	0.02101	0.23404	0.84563
Monocytes (× 10^3^/μl)	0.529 ± 0.09	0.690 ± 0.15	0.44024	0.876 ± 0.19^a^	0.01814	0.32556	0.970 ± 0.26^a^	0.00246	0.06877	0.80969
PLT (× 10^3^/μl)	855.8 ± 153.64	480.5 ± 95.76^a^	0.00034	900.667 ± 100.22^b^	0.93011	0.00009	927.5 ± 153.78^b^	0.77157	0.00004	0.98349
MPV (fL)	6.73 ± 0.08	6.81 ± 0.29	0.93814	6.78 ± 0.29	0.98543	0.99553	6.88 ± 0.26	0.73089	0.96686	0.89950
PCT (%)	0.545 ± 0.09	0.388 ± 0.14^a^	0.00870	0.610 ± 0.07^b^	0.65530	0.00062	0.635 ± 0.09^b^	0.40550	0.00023	0.97311
PDW (fL)	11.06 ± 0.26	11.70 ± 1.45	0.59472	11.26 ± 0.64	0.97782	0.82242	11.45 ± 0.64	0.86892	0.95829	0.98274

* Values are expressed as Means ± SD (n = 6). P1 represents significance difference with respect to control group (1). P2 represents significance difference with respect to Infected group (2). P3 represents significance difference with respect to Infected + ZnO NPs group (3). Differences between means were considered significant at *P* < 0.05. ^a^Significant to the control group (1), ^b^Significant to the infected group (2).

#### Biochemical parameters

A significant decrease in urea level was observed in (infected) group compared to (control) group. No significant differences were observed in creatinine, total protein, and albumin levels in any of the tested groups. However, liver enzymes GOT and GPT exhibited a nonsignificant increase in (infected) group compared to (control) group. In addition, there was a significant decrease in uric acid and glucose levels of (infected + ZnO NPs) and (ZnO NPs) groups compared to both (control) and (infected) groups (Table 3).

**Table 3:** The impact of *E. coli* infection, *E. coli* infection treated with ZnO NPs, and ZnO NPs on the biochemical parameters in female albino rats (*Rattus rattus*).

Tested parameter	Group 1 (control)	Group 2 (infected)		Group 3 (infected + ZnO NPs)			Group 4 (ZnO NPs)			
	Mean ± SD	Mean ± SD	P1	Mean ± SD	P1	P2	Mean ± SD	P1	P2	P3
Glucose (mg/dl)	106.5 ± 3.83	101.5 ± 2.34	0.43687	88.16 ± 6.49^a,b^	0.00009	0.00294	84.5 ± 8.07^a,b^	0.00001	0.00023	0.67880
Total protein (g/dl)	1.60 ± 0.21	1.61 ± 0.23	0.99936	1.47 ± 0.17	0.72734	0.65662	1.66 ± 0.23	0.96798	0.98764	0.45800
Albumin (g/dl)	3.33 ± 0.39	3.54 ± 0.35	0.92565	3.67 ± 0.43	0.75489	0.98124	4.07 ± 0.96	0.16813	0.43034	0.65251
GOT (U/l)	24.0 ± 4.51	26.2 ± 5.38	0.88791	25.8 ± 6.01	0.92790	0.99951	22.5 ± 4.80	0.95856	0.66264	0.68937
GPT (U/l)	25.6 ± 4.96	27.8 ± 2.78	0.86532	24.1 ± 6.08	0.94931	0.56770	25.6 ± 4.96	0.99871	0.86530	0.94930
Urea (mg/dl)	26.97 ± 11.08	10.52 ± 2.82^a^	0.03487	19.09 ± 7.27	0.49921	0.42923	24.90 ± 13.55	0.98150	0.07482	0.72281
Uric acid (mg/dl)	7.9 ± 0.37	7.1 ± 0.67	0.05495	4.0 ± 0.39^a,b^	0.00002	0.00002	4.2 ± 0.64^a,b^	0.00007	0.00006	0.91513
Creatinine (mg/dl)	0.365 ± 0.139	0.406 ± 0.041	0.79130	0.42 ± 0.050	0.62089	0.99075	0.385 ± 0.025	0.96998	0.96222	0.86376

*Values are expressed as Means ± SD (n = 6). P1 represents significance difference with respect to control group (1). P2 represents significance difference with respect to Infected group (2). P3 represents significance difference with respect to Infected + ZnO NPs group (3). Differences between means were considered significant at *P* < 0.05. ^a^Significant to the control group (1), ^b^Significant to the infected group (2).

### Histological sections of body organs

#### Stomach

**Fig. 5:**
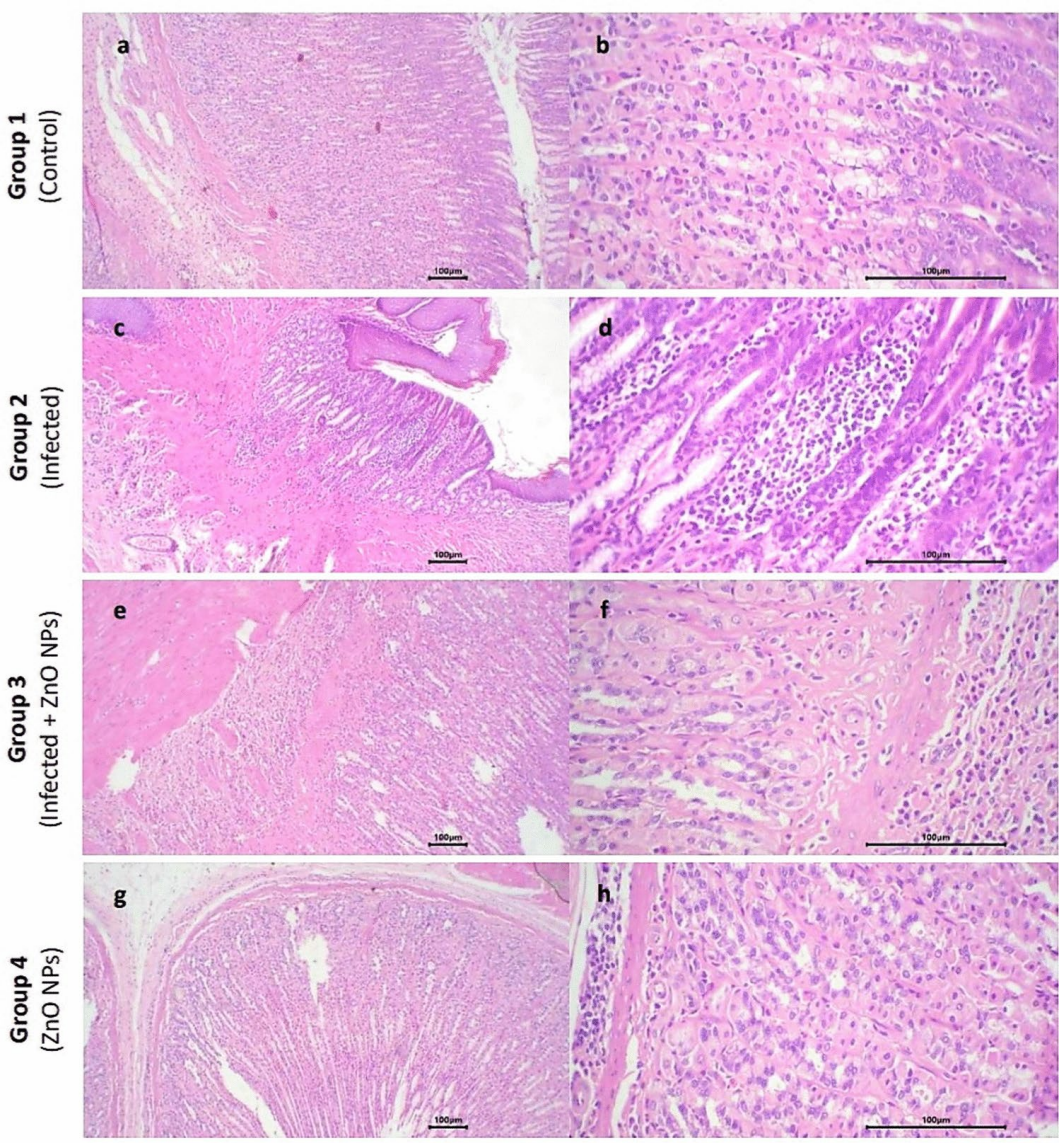
Photomicrograph of stomach sections. Group 1 exhibited (**a**) normal structure of stomach layers (× 10) and (**b**) patchy infiltration in submucosa with few congested capillaries (× 40). Group 2 exhibited (**c**) moderate infiltration in submucosa (× 10) and (**d**) inflammatory cells with multiple congested capillaries (× 40). Group 3 exhibited (**e**) normal mucosa and mucosal glands (× 10) and (**f**) mild infiltration by inflammatory cells in submucosa (× 40). Group 4 exhibited (**g**) normal mucosa and mucosal glands (× 10) and (**h**) mild infiltration by inflammatory cells in submucosa with few congested capillaries (× 40).

Gastric mucosa appeared intact with no ulceration, and the mucosal glands exhibited uniform size and shape, lined by a single layer of uniform cuboidal cells in all tested groups. However, compared to those of (control) group, the submucosa of (infected) group exhibited moderate infiltration by inflammatory cells, mainly lymphocytes, plasma cells, and eosinophils, as well as multiple congested capillaries. Conversely, the submucosa of both (infected + ZnO NPs) and (ZnO NPs) groups exhibited mild infiltration by inflammatory cells with few congested capillaries (Fig. 5).

#### Liver

**Fig. 6:**
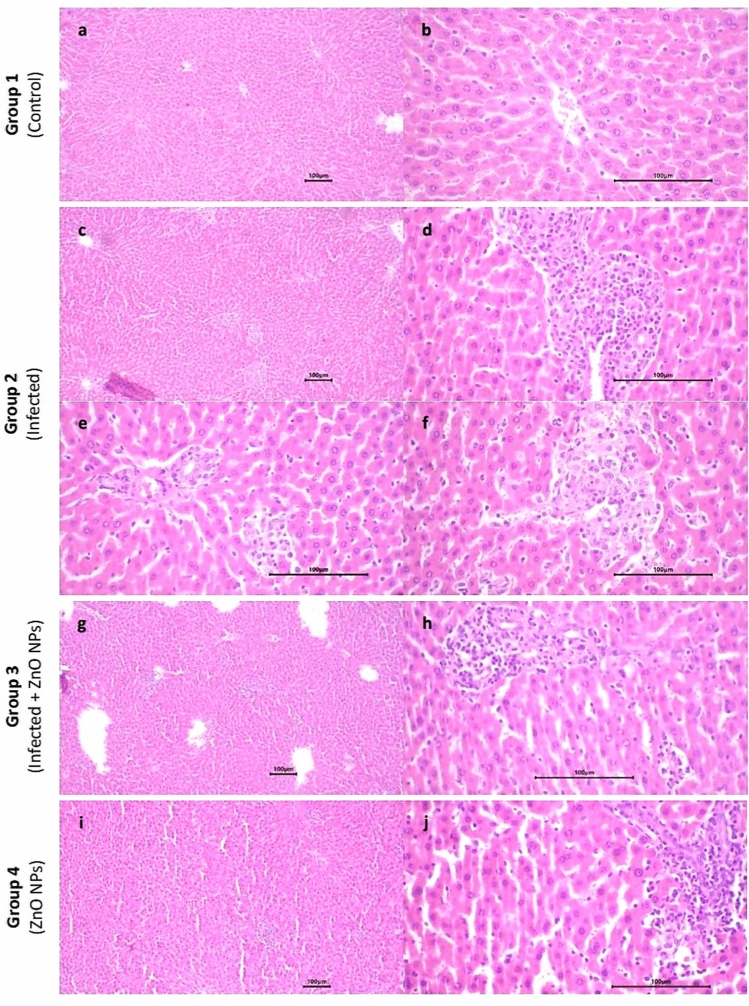
Photomicrograph of liver sections. Group 1 exhibited (**a**) normal lobular architecture of liver (× 10) and (**b**) preserved central vein, hepatocytes in a normal cording growth pattern, and normal hepatic sinusoids (× 40). Group 2 exhibited (**c**) patchy lobular inflammatory reaction and dilated central venules (× 10), (**d**) frequent inflammation of portal areas included mainly lymphocytes and plasma cells (× 40), (**e**) focal mild proliferation of bile ductules and focal congestion of hepatic sinusoids (× 40) and (**f**) focal mild hepatocyte swelling and vacuolation (× 40). Group 3 exhibited (**g**) normal lobular architecture with patchy mild lobular inflammatory reaction (× 10) and (**h**) inflammation of portal areas and mild proliferation of bile ductules (× 40). Group 4 exhibited (**i**) normal lobular architecture with no inflammatory reaction (× 10) and (**j**) minimal inflammation of portal areas (× 40).

The liver sections of all tested groups exhibited normal lobular architecture with preserved central veins and portal tracts. Focal mild hepatocyte swelling and vacuolation were observed in (infected) group compared to (control) group, while (infected + ZnO NPs) and (ZnO NPs) groups showed only swelling. Hepatic sinusoids of (infected) and (infected + ZnO NPs) groups displayed focal congestion, and the central venules were focally dilated with congestion, whereas (ZnO NPs) group exhibited normal hepatic sinusoids with no congestion and normal central venules with mild dilatation. In (infected) and (infected + ZnO NPs) groups, frequent inflammation was observed in portal areas, primarily by lymphocytes and plasma cells, along with focal mild proliferation of bile ductules and patchy lobular inflammatory reaction. Conversely, (ZnO NPs) group showed no inflammatory reaction. No steatosis or necrosis was detected in any of the tested groups (Fig. 6).

#### Kidney

**Fig. 7:**
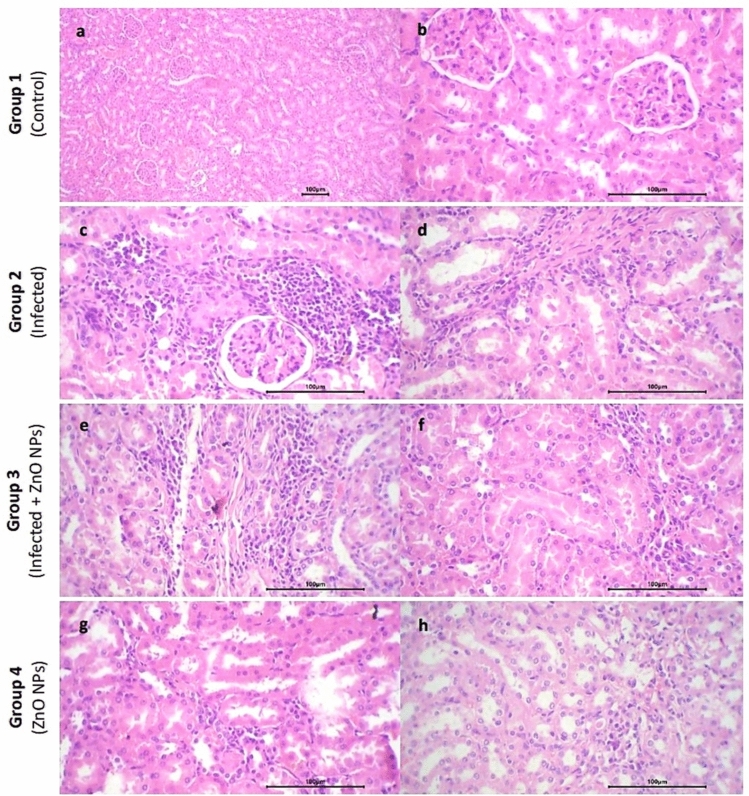
Photomicrograph of kidney sections. Group 1 exhibited (**a**) normal renal architecture (× 10) and (**b**) uniform renal tubules and normal renal glomeruli with preserved Bowman’s space (× 40). Group 2 exhibited (**c**) focal relative raised congestion of capillary glomerular tufts with multiple aggregates of inflammatory cells (× 40) and (**d**) focal congestion of capillaries (× 40). Group 3 exhibited (**e**) patchy inflammatory reaction (× 40) and (**f**) few congested stromal capillaries (× 40). Group 4 exhibited (**g**) minimal inflammatory reaction (× 40) and (**h**) few congested stromal capillaries (× 40).

The renal tissue of all tested groups exhibited normal architecture, with preserved and differentiated cortex and medulla. The renal tubules in all groups exhibited uniform size and shape, lined by a single layer of cuboidal cells with uniform nuclei, and the renal glomeruli appeared normal in size and shape with preserved Bowman’s space. However, compared to those of (control) group, the (infected) group exhibited focal relative raised congestion of capillary glomerular tufts, multiple small aggregates of inflammatory cells in renal stroma, and focal congestion of stromal capillaries. Conversely, (infected + ZnO NPs) group exhibited focal slight congestion of capillary glomerular tufts, accompanied with patchy inflammatory reaction in renal stroma with few congested stromal capillaries, while (ZnO NPs) group exhibited the minimal inflammation. No hemorrhage or necrosis was detected in any of the tested groups (Fig. 7).

#### Spleen

**Fig. 8:**
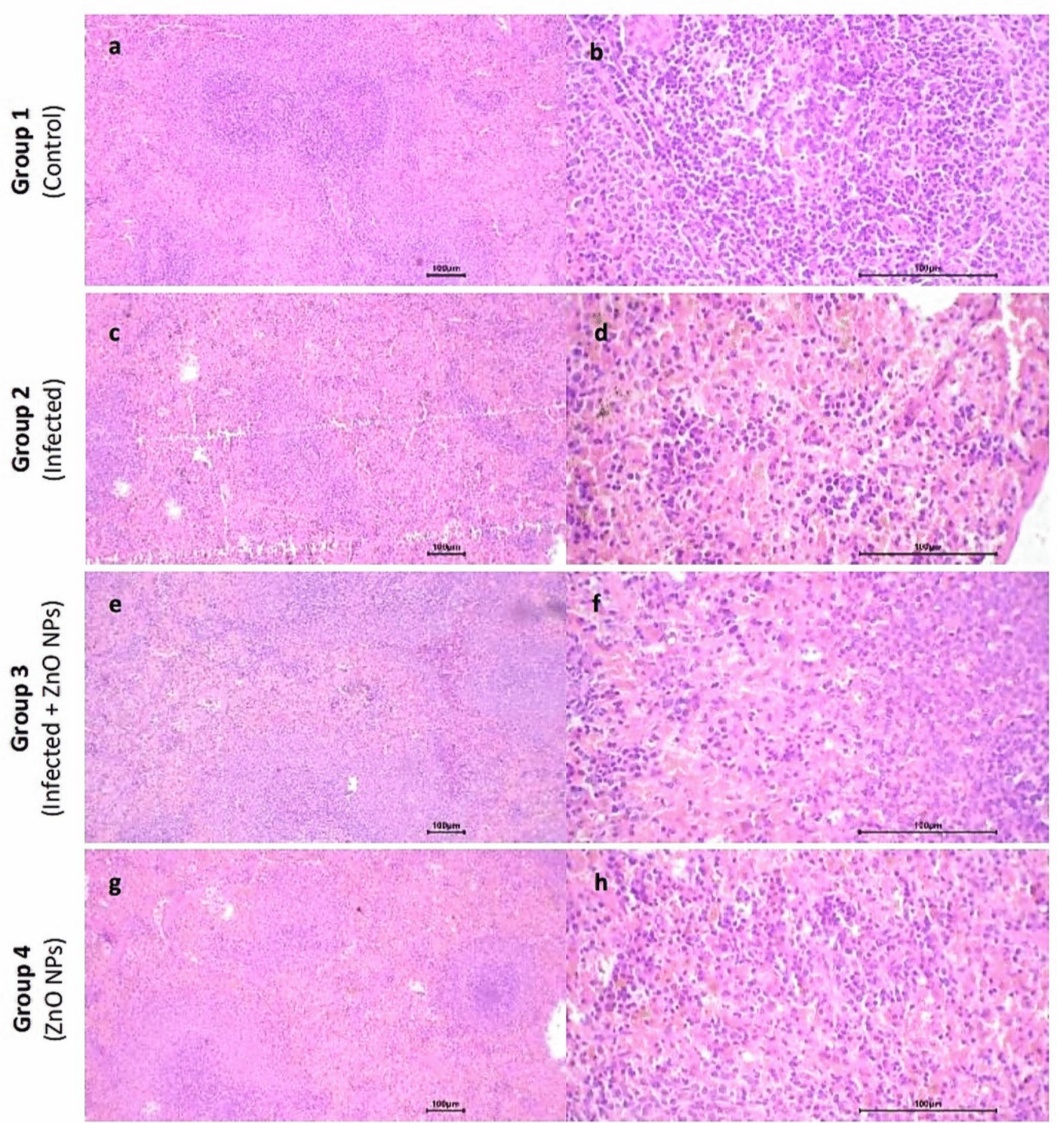
Photomicrograph of spleen sections. Group 1 exhibited (**a**) normal architecture with uniform lymphoid follicles and preserved central arterioles (× 10), and (**b**) mild inflammatory reaction in the red pulp with few congested capillaries (× 40). Group 2 exhibited (**c**) variable-sized lymphoid follicles (× 10) and (**d**) moderate inflammatory reaction in the red pulp with raised vascularity and congested capillaries (× 40). Group 3 exhibited (**e**) slightly variable-sized lymphoid follicles (× 10) and (**f**) moderate inflammatory reaction in the red pulp (× 40). Group 4 exhibited (**g**) slightly variable-sized lymphoid follicles (× 10) and (**h**) mild inflammatory reaction in the red pulp (× 40).

The spleen sections of all tested groups exhibited preserved normal architecture, with identifiable red pulp and white pulp. In (infected) group, variable-sized lymphoid follicles were observed, with a few follicles exhibiting an expanded, pale-stained center, while those in (infected + ZnO NPs) and (ZnO NPs) groups exhibited slight variability in size. The red pulp of (infected) and (infected + ZnO NPs) groups exhibited a moderate inflammatory reaction, along with increased vascularity and congested capillaries, whereas (ZnO NPs) group displayed a mild inflammatory reaction with slightly increased vascularity and scattered congested capillaries. No hemorrhage or necrosis was detected in any of the tested groups (Fig. 8).

#### Heart

**Fig. 9:**
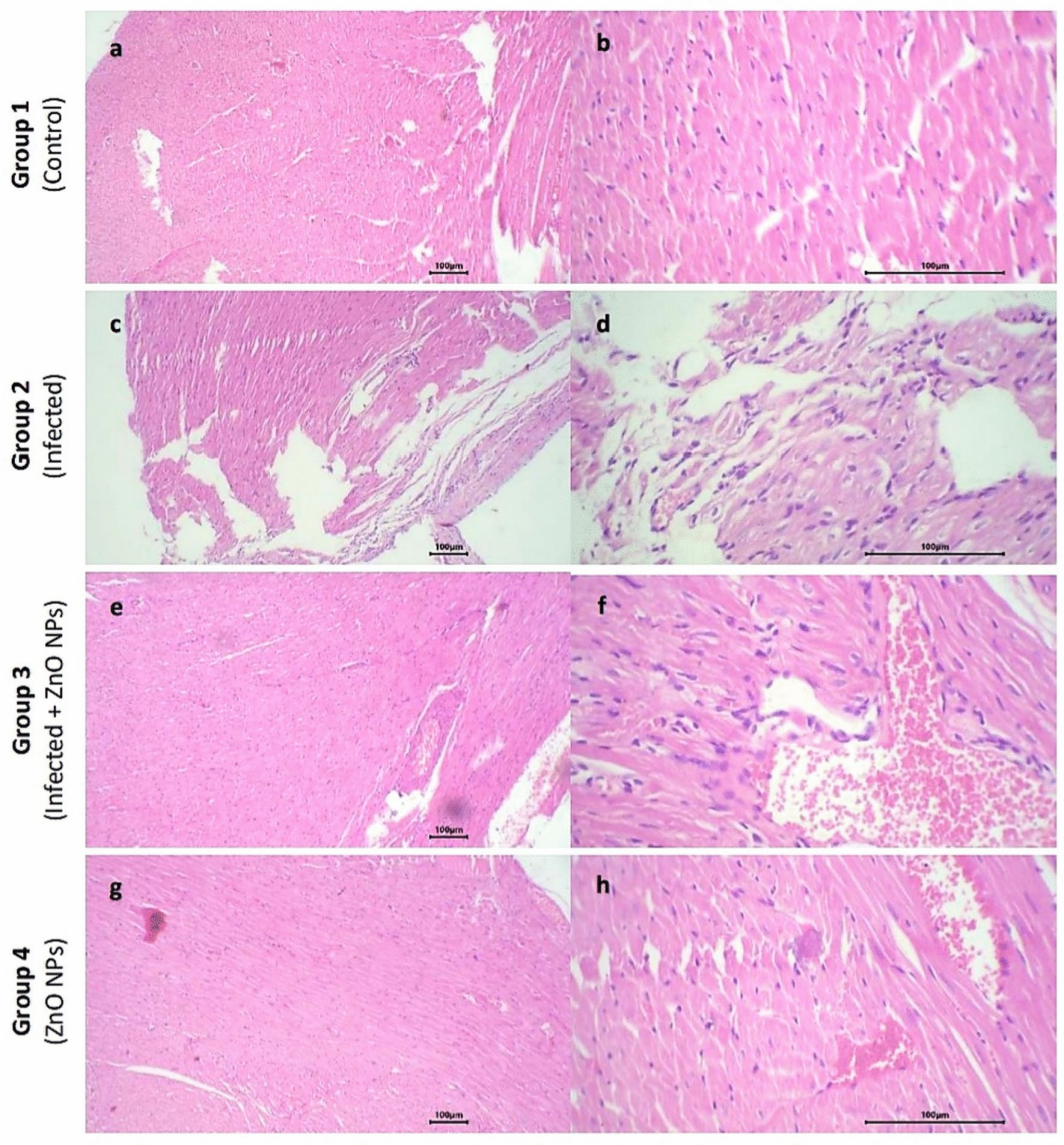
Photomicrograph of heart sections. Group 1 exhibited (**a**) normal cardiac muscle (× 10) and (**b**) uniform arrangement of muscle bundles, uniform nuclei and identified cardiac striations (× 40). Group 2 exhibited (**c**) cardiac muscle with uniform arrangement of muscle bundles (× 10) and (**d**) mild focal pericardial inflammatory reaction with few dilated capillaries (× 40). Group 3 exhibited (**e**) cardiac muscle with uniform arrangement of muscle bundles (× 10) and (**f**) few dilated capillaries with no inflammation reaction (× 40). Group 4 exhibited (**g**) cardiac muscle with uniform arrangement of muscle bundles (× 10) and (**h**) no inflammation reaction (× 40).

The cardiac muscle of all tested groups exhibited uniform arrangement of muscle bundles with consistent nuclei and identified cardiac striations. The (infected) group exhibited mild focal pericardial inflammatory reaction, mainly characterized by lymphocytes and plasma cells compared to (control) group. No inflammatory reaction was detected in either (infected + ZnO NPs) or (ZnO NPs) groups. Additionally, a few dilated capillaries were observed in (infected), (infected + ZnO NPs) and (ZnO NPs) groups. No evidence of necrosis or interstitial tissue hemorrhage was detected in any of the tested groups (Fig. 9).

## Discussion

Zinc is an essential trace element in the human body that plays a vital role in the proper functioning of macromolecules and enzymes as a coenzyme. In addition, it serves a structural function with zinc-finger structures, providing a unique scaffold for protein subdomains to interact with DNA or other proteins. Zinc is considered relatively nontoxic; however, it is possible that free zinc ions can exert negative effects on cells. To mitigate potential cytotoxicity, ZnO NPs are synthesized and bound to a ligand, rendering them nontoxic, biosafe, and biocompatible^34^. Moreover, the bioactive compounds, extracted from plants and associated with the bioreduction of ZnO NPs, could enhance their medicinal properties^35^. United States Food and Drug Administration (US FDA) has classified ZnO as a GRAS (generally recognized as safe) metal oxide^5^. ZnO NPs have gained interest as promising therapeutic agents in biology and medicine, particularly for combating issues such as ESBL-producing *E. coli* infections. In our study, the in vitro bactericidal effect of ZnO NPs was confirmed against *E. coli* OR064346, surpassing the efficacy of amoxicillin/clavulanate, the most commonly prescribed antibiotic for these infections. These findings were consistent with those of other studies reporting the antibacterial effect of ZnO NPs^12,36,37^. Gad El-Rab et al. reported that ZnO NPs could inhibit ESBL-producing uropathogens including *E. coli* and *K. pneumonia*^38^. In addition, Ahmad et al. confirmed the antimicrobial efficacy of biogenic ZnO NPs against antibiotic-resistant uropathogens^39^.

In our study, oral administration was selected as the most preferred mode of drug delivery, especially after addressing the solubility of metal oxide NPs using glycerol. Suggesting ZnO NPs as concentration-dependent bactericidal agents, we administrated a single dose (0.5 mL, 50 mg/Kg) every 24 h for 8 days of approximately 15 times of MIC for *E. coli* OR064346 (MIC = 3.2 mg/mL) and observed no abnormal signs in any of the tested groups. That was consistent with findings from Baek et al. who observed no mortality, body weight changes, or abnormal behavior upon the oral administration of (50 and 300 mg/Kg) ZnO NPs doses for 14 days^7^. Similarly, Ramadan et al. applied the oral administration of high doses (100–600 mg/Kg) of ZnO NPs for 10 weeks with no phenotypic abnormalities or death cases^25^.

In the present study, a reduction in the bacterial number was observed after the first dose of ZnO NPs administration, indicating their efficacy as a concentration-dependent drug. Similarly, Grenho et al. observed a reduction in the bacterial number after 1–3 days, using granules of hydroxyapatite and ZnO NPs in rats infected with *S. aureus*^40^. In our study, daily urine cultures confirmed the in vivo antibacterial effect of ZnO NPs, achieving a recovery percent of 83.3% after the 4th dose of ZnO NPs and a survival rate of 100% after 8 doses. These results surpassed those reported by Velumani et al. who reported that ZnO NPs treatment increased the survival rate of *P*. *aeruginosa*- and *S*. *aureus*-infected *Caenorhabditis elegans* model by 56.6% and 62.4%, respectively^41^. Rasha et al. reported improvement of wound healing in rat models infected with carbapenem-resistant *Klebsiella pneumonia* with a mean recovery percentage of 96% after 14 days of ZnO NPs application^42^. Moreover, Aboelmaaty et al. confirmed the in vivo antimicrobial efficacy of ZnO NPs against multidrug-resistant *E. coli* isolate^43^.

Bacterial infection can trigger an inflammatory response in the host, leading to abnormal coagulation cascade, disseminated intravascular coagulation (DIC) and organ failure^44^. This process can be detected through the changes in the hematopoietic system, including erythrocytes, leukocytes, and thrombocytes counts. In the present study, it was observed that the infected group experienced an erythrocytosis, as indicated by increased RBCs, HGB and HCT. Blood viscosity, represented by HCT concentration, is more biologically important than the number of circulating RBCs due to its profound role in the efficacy of tissue perfusion and hence tissue oxygenation. It is possible for blood viscosity to be normal even with a high count of RBCs if the HGB is low enough to result in a normal HCT and subsequently, in this case the HGB is low because RBCs are smaller than normal. However, it was observed that the MCV was normal in the infected group, and then the increase in RBCs and HGB would be parallel the HCT, leading to increasing of blood viscosity and a tendency to coagulation. These alterations to hematopoietic cells are mediated by the effect of microbial toxins; specifically, endotoxins, also called lipopolysaccharide (LPS) protein complexes that are localized in the outer membrane of all Gram-negative bacteria, on cellular proliferation, differentiation, and activation^45^. These LPS proteins consist of three regions; O-specific polysaccharide chain, core polysaccharide, and lipid A. Lipid A can adsorb to RBCs membranes in a tendency for bacterial iron supply from the heme molecule as an infection prerequisite causing RBC shape transformation^46^. The LPS protein was recognized as one of the most potent bacterial products involved in the induction of host inflammatory responses and tissue injury^47^. Furthermore, it was observed that all RBCs measurements were normal in ZnO NPs-administrated groups, proving the efficacy of ZnO NPs to rein the infection. It was superior to penicillins and cephalosporins that could develop hemolytic anemia after high-dosage therapy^45^. Consistent with our results, Ismail reported a significant increase of RBCs count, HGB and HCT values in *E. coli*-infected rats^48^. Similarly, Elbaz et al. revealed that *E. coli*-infected rat model exhibited a significant elevation in RBCs and HGB values^49^.

Leukocytes serve as the host’s defense against infections via a virtual mechanism called phagocytosis. In our experiment, WBCs were affected, exhibiting a leukocytosis condition, particularly in ZnO NPs-administrated groups, suggesting that ZnO NPs induced the innate response of the host. The nonsignificant increase of WBCs count observed in the infected group, was primarily attributed to the bacterial infection while the significant increase shown in ZnO NPs-administrated groups, referred to a recovery and/or an inflammatory response. Macrophages are believed to be the first cell types that process nanoparticles, mediating host inflammatory and immunological responses^50^. In our study, a significant monocytosis in the treated group indicated the recovery phase of the infection. The ability of the ZnO NPs to alter the monocytes values implied their potential as immune stimulants. Lymphocytosis shown in ZnO NPs-administrated group may refer to an inflammatory response, which was consistent with the findings of Hamouda et al. who reported that all rat groups treated with ZnO NPs exhibited an elevation of WBCs, particularly lymphocytes counts, due to an inflammatory response^51^. Similarly, Yousef et al. reported increased WBCs in the rat group treated with ZnO NPs^22^.

Platelets play a key role in maintaining the hemostasis, vascular endothelial cell integrity and wound healing. In our study, it was observed that the infected group experienced a significant decrease in the platelets count and plateletcrit. Bacterial infections are a prominent cause of diagnosed thrombocytopenia, particularly when accompanied by a slightly enlarged spleen, as observed in the infected group. Bacteria can bind to platelets either directly through a bacterial surface protein or indirectly by a plasma-bridging molecule that links bacterial and platelet surface receptor inducing platelets activation. The interaction of bacteria with platelets occurs in a diffuse manner leading to the consumption of the activated platelets, and subsequently thrombocytopenia^44,52^. Similarly, Watson et al. observed platelets activation in response to *E. coli* infection^53^. In our study, the groups treated with ZnO NPs showed a significant increase in both platelets count and plateletcrit value compared to the infected group. These results suggested the role of ZnO NPs in the earlier suppression of the possible side effects resulting from the bacterial infection.

Bacterial infection can alter the biomarkers by inducing kidney and/or liver injury, which is likely caused by cytokines, proteins induced by endotoxin of Gram-negative bacteria^54^. In the present study, it was noticed that urea levels significantly decreased in the infected group. Decreased level of serum urea induced by bacterial infection may be caused by decreased urea production (hepatic damage) and/or increased urinary urea excretion (renal damage)^55^. The treated group exhibited nonsignificant increased level of urea compared to the infected group that may refer to the recovery phase. Unlike the ZnO NPs-treated groups, it was noticed that the liver enzymes GOT and GPT exhibited a nonsignificant increase in the infected group compared to the control group that may refer to a preliminary phase of liver disease. Prakash and Chang agreed with our results that *E. coli*-infected rats could decrease the level of blood urea, suggesting that encapsulated genetically engineered live cells of *E. coli* could be given orally to lower the high urea level to normal in uremic rats^56^. On the other hand, some studies reported an increase in urea, creatinine, total protein and albumin levels in *E. coli*-infected rats^48,57^. Elbaz et al. reported a significant increase of GOT and GPT levels in rats infected with *E. coli*^49^. Al-Taii and Yousif reported that the levels of liver enzymes GOT and GPT were gradually increasing through 30 days after the infection with *E. coli*^57^.

Regarding the effect of ZnO NPs in our study, it was observed that the critical biomarkers including urea, creatinine, total protein, albumin and liver enzymes GOT and GPT exhibited normal levels in ZnO NPs-administrated groups compared to the control group. This could be a good indicator of ZnO NPs principle role in suppressing the effects of the bacterial infection. In agreement with our study, Aboelmaaty et al. demonstrated the antibacterial efficacy of plant-mediated ZnO NPs administration at a dose (50 mg/Kg) for 4 days, suggesting that a short course of ZnO NPs and administration of a suitable dose could prevent the cytotoxicity effects of the vital organs^43^. According to Ramadan et al., an elevation of urea, uric acid, GOT and GPT could be observed in groups treated with different doses of ZnO NPs (100–600 mg/Kg) for 10 weeks referring to hepatic and renal injury^25^. However, they used higher doses of chemically-synthesized ZnO NPs with larger crystal size of 30 ± 5 nm for longer period than used in the present study, which could cause the mentioned possible damage. Wang et al. agreed that larger ZnO NPs (120 nm) could induce more liver damage than small ones (20 nm) upon administration of the same dose^32^. They observed no significant differences in uric acid, creatinine, total protein, GOT or GPT levels after the administration of ZnO NPs (20 nm) at higher doses (1–5 g/Kg) for 2 weeks compared to larger ZnO NPs (120 nm). In addition, Ezealisiji et al. observed that ZnO NPs could alter the liver enzyme GPT in rats after 28 days of administration in time-dependent manner^58^.

In our study, there was a significant decrease in glucose levels in ZnO NPs-administrated groups compared to both the control and the infected groups. This indicator could indicate the potential of ZnO NPs as antidiabetic agents which may be effective for the diabetic patients, who are among the major groups predisposing to urinary tract infections. Many enzymes are activated by zinc in the body, indicating its crucial role in many metabolic pathways, including glucose metabolism. Zinc is also known to maintain the structure of insulin and plays a critical role in insulin biosynthesis, storage, secretion and signaling^59^. Several studies have confirmed that there is a correlation between zinc as a trace element and glucose metabolism and diabetes, indicating the principle role of ZnO NPs as antidiabetic agent by increasing glucose consumption and stimulation of insulin production^25,59–61^. Furthermore, in the present study, it was observed that there was a significant decrease in uric acid levels in ZnO NPs-administrated groups compared to both the control and the infected groups. This also could be a good sign, indicating the potential of ZnO NPs as anti-gouty arthritis. Similarly, many studies reported the principle role of ZnO NPs as anti-inflammatory and anti-gouty arthritis by significant decreasing of uric acid levels in blood^58,62,63^.

The stomach is the first organ affected by the oral drug delivery, and subsequently identify the fate of ZnO NPs absorption and bioavailability. In our study, it was observed that ZnO NPs-administrated group exhibited the least inflammation response compared to the other tested groups. Ensign et al. explained that when nanoparticles are orally administered, they are immobilized within the submucosal layer of the stomach and then transported into the serosal layer and systemic circulation^64^. Lee et al. managed to optically in vivo imaging of ZnO NPs following oral exposure, using infrared fluorescent technique. They reported that the nano-scaled ZnO NPs (20 nm) showed faster passage from the stomach into the gastrointestinal tract, absorption into the blood and excertion via feces compared to submicron-scaled ZnO NPs (100 nm)^65^. This could elucidate the mild effect of the small-sized ZnO NPs (20 nm) in our study. Wang et al. and Pasupuleti et al. reported moderate inflammatory cell infiltration in rat stomach due to administration of ZnO NPs^32,66^. Conversely, in our study, the most adverse inflammatory reaction observed in the infected group may be attributed to systemic inflammatory response induced by LPS of Gram-negative bacteria. Brown et al. reported that LPS could induce the activation of nitric oxide synthase in rat gastric mucosal cells^67^. Induction of this enzyme contributes to inflammation, cellular damage and endotoxic shock.

The liver is a pivotal body organ that is responsible for detoxification^68^. The liver and spleen together constitute the integral parts of fixed macrophages called the reticuloendothelial system that could filter bacteria and other foreign particles from the bloodstream^69^. The liver could modulate the systemic response to severe infection via Kupffer cells that can clear the endotoxins and bacteria, initiating the systemic inflammatory response. At the same time, Kupffer cells and hepatocytes may get activated by of bacteria and endotoxins and become sources of soluble mediators of systemic inflammatory response that may lead to focal cellular injury^70^. In our study, the bacterial infection in the infected group adversely induced acute liver injury compared to the control and ZnO NPs-administrated groups. In agreement with our observations, Elbaz et al. observed that *E.coli*-infected rat group showed intense lymphocytic infiltration in the hepatic lobules^49^. Radwan et al. reported that *E. coli*-infected rats showed aggressive alterations including congestion of central, portal veins and hepatic sinusoids, hemorrhage in the hepatic parenchyma, hyperplastic proliferation of biliary epithelium, and multifocal degenerative changes with extensive coagulative necrosis^71^. Aboelmaaty et al. reported that the administration of *E. coli*-LPS induced multifocal leukocytic cells infiltration, severe hemorrhage, vacuolar degeneration, and necrosis of hepatocytes in rat liver, unlike ZnO NPs-treated rats, suggesting the protective role of ZnO NPs as antibacterial and anti-inflammatory agents^43^. Abd El Megid et al. confirmed that the liver showed normal structure after 5 days of ZnO NPs administration^72^. In addition, Sohail et al. reported that no structural changes were observed in rats liver administrated to Neem-based ZnO NPs for 14 days, suggesting the biosafety of green-synthesized ZnO NPs as antibacterial agents^73^. Hatab et al. reported that dietary supplementation of the biosynthesized ZnO NPs (60 mg/Kg) exhibited normal liver architecture and minimal alterations detected after 5 weeks^74^. According to Ramadan et al. who reported that the effects of ZnO NPs-administration could be induced in a dose manner, it was observed that no adverse effects were induced in rat liver in groups treated with 100–300 mg/Kg, however, alterations, including hepatic vacuolation, large sinusoidal dilatation, degenerative alterations, and cellular congestion, were induced in rats treated with 400–600 mg/Kg^25^.

The kidney is considered among the major vital organs that could be affected by bacterial infection, particularly urinary tract infections, as well as being involved in the biodistribution of nanoparticles in the body^68^. The LPS of bacteria can directly induce an acute kidney injury^75^. In the present study, the infected group exhibited acute kidney injury compared to the control and ZnO NPs-administrated groups. Wang et al. confirmed that the virulence factors associated with uropathogenic *E. coli* can mediate kidney injury and acute pyelonephritis and may also pave to permanent renal scarring and failure^76^. In addition, Radwan et al. reported that rats infected with *E. coli* exhibited severe kidney damage, including marked hyperemia, expanded cortical interstitium, interstitial infiltrations with hemorrhage, and coagulative necrosis^71^. Aboelmaaty et al. reported that the histopathological changes showed massive damage to liver and kidney of *E. coli* LPS-treated rats, which was reversed to a great extent by ZnO NPs administration^43^. Wang et al. observed that there was no considerable nephrotoxicity in rats after ZnO NPs administration^32^. Furthermore, Barakat et al. revealed that ZnO NPs exhibited a protective role as anti-inflammatory agents in rats with kidney injury that was caused by cisplatin^27^. Sohail et al. observed no considerable histological alteration in kidneys of green ZnO NPs-treated rats^73^. Hamouda et al. reported that ZnO NPs administration for the treatment of bacterial infection decreased the severity of histological alterations but did not return the tissue to normal case, suggesting that biologically-derived ZnO NPs are more effective than chemically-synthesized ZnO NPs^51^. Yousef et al. reported that ZnO NPs (chemically synthesized, 100 nm, 100 mg/Kg) could induce hepatorenal toxicity in rats^22^.

Splenic architecture is well-suited for performing two crucial functions: phagocytosis and antibody production, allowing an efficient immune response^69^. Zinc and other trace elements that mainly contribute to regulation of the immune response, are significantly decreased due to bacterial infection and endotoxin challenges. Zinc deficiency may lead to decreased antibody formation, impaired T-cell response, and lymphoid atrophy^77^. In the present study, the infected group exhibited slightly enlarged spleen and histologically showed a moderate inflammatory reaction compared to the control and ZnO NPs-administrated groups. Similarly, Radwan et al. observed a marked congestion of splenic blood vessels with perivascular inflammatory cellular aggregates in rats infected with *E. coli*^71^. Shah et al. reported that the inoculation of rats with of *E. coli* could induce severe leukocytic infiltration and hemorrhage on the capsular spleen surface^78^. In our study, ZnO NPs-administrated groups showed the minimal alterations of spleen. In agreement with our results, Singh et al. observed no considerable alterations after ZnO NPs (50 mg/Kg) administration for 4 weeks, however, congestion of red pulp and widened sinusoids were observed in case of high dose (250 mg/Kg) referring to the importance of detection of dose-related effects^79^. In addition, Wang et al. reported that ZnO NPs could induce the lessened possible damage to the vital organs, in particular spleen, in a size-dependent manner where ZnO NPs of 20 nm in size showed no damage versus 120 nm-sized ones^32^.

The LPS of Gram-negative bacteria could induce aggressive effects on several organs as shown, including the heart, leading to multiple organ failure and death. As a high-energy expenditure organ, the heart is particularly sensitive to the LPS-induced oxidative damage^80^. In the present study, the infected group exhibited a marked pericardial inflammation compared to the control and ZnO NPs-administrated groups. Radwan et al. reported that the rats infected with *E. coli* exhibited an inflammatory reaction associated with hemorrhages and necrosis in the myocardium^71^. In our study, it was observed that ZnO NPs-administration effectively suppressed the inflammation of cardiac tissue, suggesting that their pivotal potential as antioxidant agents may be included in cardio-protection. Our results were in accordance with Rezaei et al. who reported that ZnO NPs could induce cardiomyocyte recovery with restoration of the normal architecture of the muscle fibers and significant reduction of monocyte infiltration in diabetic rats^81^. In contrast, other studies reported that ZnO NPs administration induced an inflammatory reaction in rats heart^66,82^. Abdel-Magied and Shedid observed that ZnO NPs played a vital protective role to the cardiovascular tissue in a dose-dependent manner where a low dose (10 mg/Kg) mitigated the oxidative stress and inflammation effects, and a high dose (300 mg/Kg) induced cardiovascular toxicity of rats^83^. Similarly, Adamu et al. observed a healing in cardiac tissue with no considerable toxicity by dose-dependent administration of green-synthesized ZnO NPs in hyperlipidemic rats^84^.

## Conclusion

Biosynthesized ZnO NPs are considered promising therapeutic agents. The present study addressed the antibacterial impact of ZnO NPs and their principle role, suppressing the effects of the bacterial infection. Based on our study, it is suggested that the oral administration at a dose of 50 mg/Kg of the biosynthesized ZnO NPs with 20 nm-size for short period could exhibit an antibacterial efficacy against uropathogenic *E. coli* with no considerable toxicity to vital body organs.

## Materials and methods

### Ethical approval

The study received Ethical approval from Committee for Scientific Research Ethics, Faculty of Science, Sohag University, Egypt (CSRE-13-24).

### Animal guidelines

The study is reported in accordance with ARRIVE guidelines. All methods were carried out in accordance with relevant guidelines and regulations.

### Bacterial strain and culture conditions

The bacterial strain was isolated from urine sample of UTI-confirmed case (20 years-old-male with pyuria and UTI symptoms), cultured on cysteine lactose electrolyte-deficient agar, (CLED agar, w/Bromo Thymol Blue M792-HiMedia Laboratories, India) at 35±2°C for 24 h and preliminary identified with Gram staining and biochemical tests^2^. Detection of extended spectrum β-lactamase (ESBL) was performed based on the criteria described by Clinical Laboratory Standards Institute (CLSI) published document^85^. The strain was cultured in nutrient broth medium^86^ and incubated at 28°C for 48 h prior to being sent to The Molecular Biology Research Unit, Assiut University, Egypt for DNA extraction. Patho-gene-spin DNA/RNA extraction kit provided by Intron Biotechnology Company, Korea was used. The extracted DNA was shipped to SolGent Company, Daejeon South Korea for polymerase chain reaction (PCR) and 16S gene sequencing. PCR was performed using two universal primers namely 27F (5′-AGAGTTTGATCCTGGCTCAG-3′) and 1492R (5′-GGTTACCTTGTTACGACTT-3′). The purified PCR product was confirmed using a size nucleotide marker (100 base pairs) by electrophoreses on 1% agarose gel. Purified product was sequenced in the sense and antisense directions using 27F and 1492R primers with the incorporation of dideoxy nucleotides (dd NTPs) in the reaction mixture^87^. Sequences were further analyzed using Basic Local Alignment Search Tool (BLAST) from the National Center of Biotechnology Information (NCBI) website. Phylogenetic analysis of sequences was done using MegAlign (DNA Star) software version 5.05.

### Biosynthesis and characterization of ZnO NPs

Zinc oxide nanoparticles were biosynthesized using *Aloe vera* leaves extract according to Abu-Gharbia et al.^33^ and were detected by V-770 UV–Vis/NIR Spectrophotometer (Jasco, UK) at wavelength range between 200 and 800 nm. X-ray diffraction (XRD) analyzer (Bruker D8 Advance, Germany) provided information interpreting the crystalline structure, lattice parameters and crystalline grain size of ZnO NPs. Morphology and median particle size of ZnO NPs were provided by transmission electron microscopy (The Electron Microscopy Unit, Assuit University, Egypt).

### Testing in vitro antibacterial impact of ZnO NPs

The in vitro antibacterial impact of ZnO NPs was tested using disk diffusion susceptibility test. Briefly, a 24-h bacterial growth was adjusted to a 0.5 McFarland standard and inoculated on Mueller Hinton agar (MHA) plates. Definite concentration of ZnO NPs (5 mg/mL) was prepared by dissolving in glycerol and water (4:1 v/v, respectively) and sonicated by sonicator Q700 (Terra universal, USA) for 10 min. Sterilized filter paper disc (5 mm) was loaded by 100 μL of ZnO NPs and put on the MHA plates. Amoxicillin/clavulanate (AMC; 20/10 μg) disc (Microxpress-Tulip Diagnostics Labs, India) was used as a positive control. Another disc was loaded by glycerol and water (4:1 v/v, respectively) and used as a negative control. The plates were incubated at 35±2°C for 24 h. The minimum inhibitory concentration (MIC) and the minimum bactericidal concentration (MBC) were detected by the broth microdilution assay using 96-well microtiter plate based on the criteria described by CLSI published document^88^.

### Testing in vivo antibacterial impact of ZnO NPs on rat model

#### Animals and experimental groups

Twenty four female albino rats (*Rattus rattus*), 2–3 months age and weighing 180 g were used in the study. Animals were obtained from Zoology Department, Faculty of Science, Sohag University, Egypt and then they were housed in cages and kept on a standard diet. They were maintained in a controlled atmosphere, a temperature of 25±2°C and 55% humidity. After 10 days of acclimation, animals were divided into four equal groups as follows:

A. Group 1: control group, received 0.2 mL of solvent (glycerol and water) as nanoparticles vehicle.
B. Group 2: infected with *E. coli* OR064346 (0.5 mL of 0.5 MacFarland standard) by intraperitoneal injection.
C. Group 3: infected with *E. coli* OR064346 (0.5 mL of 0.5 MacFarland standard) by intraperitoneal injection and after 24 h of infection induction, the rats were applied daily to oral administration of 0.5 mL of ZnO NPs (50 mg/Kg) for 8 days.
D. Group 4: applied daily to oral administration of 0.5 mL of ZnO NPs (50 mg/Kg) for 8 days.

Urine cultures were performed daily on CLED agar with Andrade indicator (Accumix-Tulip Diagnostics Labs, India) to indicate the progress of infection and the effect of ZnO NPs as antibacterial agents. At the end of the experiment, animals groups were sacrificed (euthanasia; decapitation).

### Blood samples collection

Blood samples were collected from rats and transferred to two types of tubes: first, tubes containing EDTA as an anticoagulant (for the hematological analysis) and second, plain tubes (for the biochemical analysis). The collected blood in plain tubes was centrifuged at 2000 rpm for 20 min to separate the serum for the analysis of tested parameters.

#### Hematological parameters

Auto-hematology analyzer (RAYTO RT-7600) was used to perform a complete blood count (CBC) for each rat, measuring the following parameters: red blood cells (RBCs) count, hemoglobin (HGB), hematocrit (HCT) value, mean corpuscular volume (MCV), mean corpuscular hemoglobin (MCH), mean corpuscular hemoglobin concentration (MCHC), red blood cell distribution width-coefficient of variation (RDW-CV), white blood cells (WBCs) count, platelets (PLT) count, mean platelet volume (MPV), plateletcrit (PCT) and platelet distribution width (PDW).

#### Biochemical parameters

Renal function biomarkers including urea, uric acid and creatinine, and glucose were measured by colorimetric kits that were provided from Spectrum Diagnostics, Egypt. Liver function biomarkers comprising total protein and albumin were measured by colorimetric kits that were provided from Spectrum Diagnostics, Egypt. Liver enzymes including glutamic oxaloacetic transaminase (GOT) and glutamyl pyruvic transaminase (GPT) were measured kinetically using kits from Chema Diagonstica, Italy. All biomarkers were conducted on the serum according to manufacturer’s instructions.

### Histological sections of body organs

Vital organs (stomach, liver, kidney, spleen, and heart) were obtained from rats, washed with running tap water for 2 h, fixed in 10% neutral buffered formaldehyde, and then treated with a conventional grade of ethyl alcohol 96% (v/v) and xylol. After that, they were embedded in the paraffin and sectioned at 4–6 μm thickness. The sections were stained with Haematoxylin and Eosin stains and photographed using a light microscope.

### Statistical analysis

Data of the in vivo study (the hematological and biochemical parameters) were presented as means ± standard deviation (SD) according to the method reported by Snedecor and Cochran^89^. Analysis of variance (ANOVA) was performed using the software SPSS 16. The level of significance was measured running a Takey test; values at *P* < 0.05 were statistically considered as significant.

## Data Availability

The 16S rRNA gene sequence of the bacterial isolate was deposited in the GenBank with accession number *Escherichia coli* OR064346 (https://www.ncbi.nlm.nih.gov/nuccore/2512327141). Other data supporting the findings of this study are not publicly available (due to the intellectual property rights as it is a part of MSc thesis belongs to G.A.) but are available from the corresponding author on a reasonable request.

## Declarations

### Dual Publication

Figure 1A, exhibiting *Escherichia coli* OR064346 strain appearance on CLED agar, and Fig. 2, exhibiting characterization of the biosynthesized ZnO NPs, are reproduced from our previous publications (References 2 and 33, respectively).

## References

[1] Dadi, B. et al. Distribution of virulence genes and phylogenetics of uropathogenic *Escherichia coli* among urinary tract infection patients in Addis Ababa, Ethiopia. *BMC Infect. Dis.* **20**, 108. 10.1186/s12879-020-4844-z (2020). 32033541 10.1186/s12879-020-4844-z PMC7006406

[2] Abu-Gharbia, M. A., Al-Arabi, G. & Salem, J. M. Community-acquired urinary tract infections: Epidemiology, etiology, and β-lactam resistance. *Sohag J. Sci.* **9**, 47–55. 10.21608/sjsci.2023.226098.1103 (2024).

[3] Levy, S. B. & Marshall, B. Antibacterial resistance worldwide: Causes, challenges and responses. *Nat. Med.* **10**, 122–129. 10.1038/nm1145 (2004). 10.1038/nm1145 15577930

[4] Rudramurthy, G. R., Swamy, M. K., Sinniah, U. R. & Ghasemzadeh, A. Nanoparticles: Alternatives against drug-resistant pathogenic microbes. *Molecules* **21**, 836–866. 10.3390/molecules21070836 (2016). 27355939 10.3390/molecules21070836 PMC6273897

[5] Agarwal, H., Kumar, S. V. & Rajeshkumar, S. A review on green synthesis of zinc oxide nanoparticles: An eco-friendly approach. *Res. Eff. Technol.* **3**, 406–413. 10.1016/j.reffit.2017.03.002 (2017).

[6] Reddy, L. S., Nisha, M. M., Joice, M. & Shilpa, P. N. Antimicrobial activity of zinc oxide (ZnO) nanoparticle against *Klebsiella pneumoniae*. *Pharm. Biol.* **52**, 1388–1397. 10.3109/13880209.2014.893001 (2014). 25026353 10.3109/13880209.2014.893001

[7] Baek, M. et al. Pharmacokinetics, tissue distribution, and excretion of zinc oxide nanoparticles. *Int. J. Nanomed.* **7**, 3081–3097. 10.2147/IJN.S32593 (2012). 10.2147/IJN.S32593 PMC3394467 22811602

[8] Gunalan, S., Sivaraja, R. & Rajendran, V. Green synthesized ZnO nanoparticles against bacterial and fungal pathogens. *Prog. Nat. Sci.* **22**, 693–700. 10.1016/j.pnsc.2012.11.015 (2012).

[9] Umar, H. Morphological changes caused by synthesized zinc oxide nanoparticles in MDA-MB 231 cells and prediction with multi-linear regression. *Trop. J. Nat. Prod. Res.* **7**, 5616–5622 (2023).

[10] Suresh, D. et al. EGCG assisted green synthesis of ZnO nanopowders: Photodegradative, antimicrobial and antioxidant activities. *Spectrochim. Acta A Mol. Biomol.* **136**, 1467–1474. 10.1016/j.saa.2014.10.038 (2015). 10.1016/j.saa.2014.10.038 25459708

[11] Huang, Y., Wu, C. & Aronstam, R. S. Toxicity of transition metal oxide nanoparticles: Recent insights from *in-vitro* studies. *Materials* **3**, 4842–4859. 10.3390/ma3104842 (2010). 28883356 10.3390/ma3104842 PMC5445783

[12] Ali, K. et al. *Aloe vera* extract functionalized zinc oxide nanoparticles as nano antibiotics against multi-drug resistant clinical bacterial isolates. *J. Coll. Interface Sci.* **472**, 145–156. 10.1016/j.jcis.2016.03.021 (2016). 10.1016/j.jcis.2016.03.021 27031596

[13] Anand, G. T. et al. Green synthesis of ZnO nanoparticle using *Prunus dulcis* (almond gum) for antimicrobial and supercapacitor applications. *Surf. Interfaces* **17**, 100376. 10.1016/j.surfin.2019.100376 (2019).

[14] Batool, M., Khurshid, S., Qureshi, Z. & Daoush, W. M. Adsorption, antimicrobial and wound healing activities of biosynthesized zinc oxide nanoparticles. *Chem. Pap.* **75**, 893–907. 10.1007/s11696-020-01343-7 (2021).

[15] Singh, J. et al. Green synthesis of metals and their oxide nanoparticles: Applications for environmental remediation. *J. Nanobiotechnol.* **16**, 84. 10.1186/s12951-018-0408-4 (2018). 10.1186/s12951-018-0408-4 PMC6206834 30373622

[16] Gupta, D., Boora, A., Thakur, A. & Gupta, T. Green and sustainable synthesis of nanomaterials: Recent advancements and limitations. *Environ. Res.* **231**, 116316. 10.1016/j.envres.2023.116316 (2023). 37270084 10.1016/j.envres.2023.116316

[17] Gudikandula, K. & Maringanti, S. C. Synthesis of silver nanoparticles by chemical and biological methods and their antimicrobial properties. *J. Exp. Nanosci.* **11**, 714–721. 10.1080/17458080.2016.1139196 (2016).

[18] Cowan, M. M. Plant products as antimicrobial agents. *Clin. Microbiol. Rev.* **12**, 564–582. 10.1128/cmr.12.4.564 (1999). 10515903 10.1128/cmr.12.4.564 PMC88925

[19] Brayner, R. et al. Toxicological impact studies based on *Escherichia coli* bacteria in ultrafine ZnO nanoparticles colloidal medium. *Nano Lett.* **6**, 866–870. 10.1021/nl052326h (2006). 16608300 10.1021/nl052326h

[20] Xia, T. et al. Comparison of the mechanism of toxicity of zinc oxide and cerium oxide nanoparticles based on dissolution and oxidative stress properties. *ACS Nano* **2**, 2121–2134. 10.1021/nn800511k (2008). 19206459 10.1021/nn800511k PMC3959800

[21] Horky, P. et al. Zinc phosphate-based nanoparticles as a novel antibacterial agent: In vivo study on rats after dietary exposure. *J. Anim. Sci. Biotechnol.* **10**, 17. 10.1186/s40104-019-0319-8 (2019). 30805185 10.1186/s40104-019-0319-8 PMC6373129

[22] Yousef, M., Mutar, T. & Kamel, M. Hepato-renal toxicity of oral sub-chronic exposure to aluminum oxide and/or zinc oxide nanoparticles in rats. *Toxicol. Rep.* **6**, 336–346. 10.1016/j.toxrep.2019.04.003 (2019). 31049295 10.1016/j.toxrep.2019.04.003 PMC6482313

[23] Rahdar, A., Hajinezhad, M. R., Bilal, M., Askari, F. & Kyzas, G. Z. Behavioral effects of zinc oxide nanoparticles on the brain of rats. *Inorg. Chem. Commun.* **119**, 108131. 10.1016/j.inoche.2020.108131 (2020).

[24] Kavaz, D., Abubakar, D. L., Rizaner, N. & Umar, H. Biosynthesized ZnO nanoparticles using *Albizia lebbeck* extract induced biochemical and morphological alterations in wistar rats. *Molecules* **26**, 3864. 10.3390/molecules26133864 (2021). 34202852 10.3390/molecules26133864 PMC8270351

[25] Ramadan, A., Yassein, A., Eissa, E., Mahmoud, M. & Hassan, G. Biochemical and histopathological alterations induced by sub chronic exposure to zinc oxide nanoparticle in male rats and assessment of its genotoxicity. *J. Umm Al-Qura Univ. Appl. Sci.* **8**, 41–49. 10.1007/s43994-022-00008-3 (2022).

[26] Song, W. et al. Role of the dissolved zinc ion and reactive oxygen species in cytotoxicity of ZnO nanoparticles. *Toxicol. Lett.* **199**, 389–397. 10.1016/j.toxlet.2010.10.003 (2010). 20934491 10.1016/j.toxlet.2010.10.003

[27] Barakat, L., Barakat, N., Zakaria, M. & Khirallah, S. Protective role of zinc oxide nanoparticles in kidney injury induced by cisplatin in rats. *Life Sci.* **262**, 118503. 10.1016/j.lfs.2020.118503 (2018). 10.1016/j.lfs.2020.118503 33007311

[28] El-Maddawy, Z. K. & Abd El Naby, W. S. H. Protective effects of zinc oxide nanoparticles against doxorubicin induced testicular toxicity and DNA damage in male rats. *Toxicol. Res.* **8**(5), 654–662. 10.1039/c9tx00052f (2019). 10.1039/c9tx00052f PMC6762007 31588342

[29] Mohamed, D. A. & Abdelrahman, S. A. The possible protective role of zinc oxide nanoparticles (ZnO NPs) on testicular and epididymal structure and sperm parameters in nicotine-treated adult rats (a histological and biochemical study). *Cell Tissue Res.* **375**, 543–558. 10.1007/s00441-018-2909-8 (2019). 30218240 10.1007/s00441-018-2909-8

[30] Ghareeb, O. A. Toxicopathological effects of zinc oxide nanoparticles on the liver function and preventive role of silymarin in vivo. *Indian J. Forensic Med. Toxicol.* **15**, 3212–3217. 10.37506/ijfmt.v15i2.14863 (2021).

[31] Chang, Y.-N., Zhang, M., Xia, L., Zhang, J. & Xing, G. The toxic effects and mechanisms of CuO and ZnO nanoparticles. *Materials* **5**(12), 2850–2871. 10.3390/ma5122850 (2012).

[32] Wang, B. et al. Acute toxicological impact of nano- and sub micro-scaled zinc oxide powder on healthy adult mice. *J. Nanopart. Res.* **10**, 263–276. 10.1007/s11051-007-9245-3 (2008).

[33] Abu-Gharbia, M. A., Salem, J. M. & Al-Arabi, G. Biosynthesis of zinc oxide nanoparticles using *Aloe vera* leaves extract and their antibacterial impact. *Bull. Pharm. Sci. Ass. Univ.* **47**, 499–517. 10.21608/bfsa.2023.239425.1934 (2024).

[34] Gudkov, S. V. et al. A mini review of antibacterial properties of ZnO nanoparticles. *Front. Phys.* **9**, 641481. 10.3389/fphy.2021.641481 (2021).

[35] Ainyanbhor, I. E. et al. Acute and sub-acute toxicity study of aqueous and methanol root extract of *Tetracera alnifolia* in male albino rats. *Toxicol. Rep.* **13**, 101786. 10.1016/j.toxrep.2024.101786 (2024). 39526239 10.1016/j.toxrep.2024.101786 PMC11543917

[36] Mustafa, S. et al. Effect of ZnO nanoparticles on ESBL producing *Escherichia coli* & *Klebsiella* spp. *Eastern J. Med.* **16**, 253–257 (2011).

[37] Maruthupandy, M., Rajivgandhi, G., Muneeswaran, T. & Song, J. Biologically synthesized zinc oxide nanoparticles as nano antibiotics against ESBLs producing Gram negative bacteria. *Microb. Pathog.* **121**, 224–231. 10.1016/j.micpath.2018.05.041 (2018). 29807135 10.1016/j.micpath.2018.05.041

[38] Gad El-Rab, S. M., Abo-Amer, A. E. & Asiri, A. M. Biogenic synthesis of ZnO nanoparticles and its potential use as antimicrobial agent against multidrug-resistant pathogens. *Curr. Microbiol.* **77**, 1767–1779. 10.1007/s00284-020-01991-8 (2020). 32328748 10.1007/s00284-020-01991-8

[39] Ahmad, N. et al. Antimicrobial efficacy of *Mentha piperata*-derived biogenic zinc oxide nanoparticles against UTI-resistant pathogens. *Sci. Rep.* **13**, 14972. 10.1038/s41598-023-41502-w (2023). 37696980 10.1038/s41598-023-41502-w PMC10495404

[40] Grenho, L., Salgado, C. L., Fernandes, M. H., Monteiro, J. F. & Ferraz, M. P. Antibacterial activity and biocompatibility of three-dimensional nanostructured porous granules of hydroxyapatite and zinc oxide nanoparticles-an in vitro and in vivo study. *Nanotechnology* **26**, 315101. 10.1088/0957-4484/26/31/315101 (2015). 26180062 10.1088/0957-4484/26/31/315101

[41] Velumani, M. et al. Green synthesis of zinc oxide nanoparticles using *Cananga odorata* essential oil and its antibacterial efficacy in vitro and in vivo. *Comp. Biochem. Physiol. C Toxicol. Pharmacol.* **26**, 109448. 10.1016/j.cbpc.2022.109448 (2022). 10.1016/j.cbpc.2022.109448 36064134

[42] Rasha, E. et al. Effects of zinc oxide nanoparticles synthesized using *Aspergillus niger* on carbapenem-resistant *Klebsiella pneumonia *in vitro and in vivo. *Front. Cell. Infect. Microbiol.* **11**, 748739. 10.3389/fcimb.2021.748739 (2021). 34869059 10.3389/fcimb.2021.748739 PMC8635236

[43] Aboelmaaty, A., Omara, S., Aly, M., Kotp, M. & Ali, A. The antibacterial and anti-inflammatory effects of zinc oxide nanoparticles synthesized by *Thymus vulgaris* medicinal plant against *Escherichia coli* and *Escherichia coli* lipopolysaccharides. *Egypt. Pharm. J.* **21**, 153–166. 10.4103/epj.epj_98_21 (2022).

[44] Fitzgerald, J. R., Foster, T. J. & Cox, D. The interaction of bacterial pathogens with platelets. *Nat. Rev. Microbiol.* **4**, 445–457. 10.1038/nrmicro1425 (2006). 16710325 10.1038/nrmicro1425

[45] Beck, N. *Diagnostic hematology* (Springer-Verlag London Limited, Heidelberg, 2009). 10.1007/978-1-84800-295-1.pdf.

[46] Poschl, M. B., Ruef, P., Schnauffer, M. & Linderkantp, O. The effect of different *Escherichia coli* endotoxins on red blood cell deformabdity. *Clin. Hemorheol. Microcirc.* **15**, 749–753 (1995).

[47] Al-Sagair, O. A., El-Daly, E. S. & Mousa, A. Influence of bacterial endotoxins on bone marrow and blood components. *Med. J. Islamic World Acad. Sci.* **17**, 23–36 (2009).

[48] Ismail, H. The ameliorative efficacy of *Thymus vulgaris* essential oil against *Escherichia coli* O157:H7-induced hematological alterations, hepatorenal dysfunction and immune-inflammatory disturbances in experimentally infected rats. *Environ. Sci. Pollut. Res. Int.* **29**, 41476–41491. 10.1007/s11356-022-18896-7 (2022). 35088282 10.1007/s11356-022-18896-7

[49] Elbaz, H., Hamed, M., Abdelhamid, F. & Abdalla, O. Effect of cefepime on hematological, immunological and oxidant/antioxidant parameters in rats experimentally infected with *E.**coli* ATCC 25922. *Mansoura Vet. Med. J.* **21**, 36–45. 10.35943/mvmj.2020.21.116 (2020).

[50] Gustafson, H., Casper, D., Grainger, D. & Ghandehari, H. Nanoparticle uptake: The phagocyte problem. *Nano Today* **10**, 487–510. 10.1016/j.nantod.2015.06.006 (2015). 26640510 10.1016/j.nantod.2015.06.006 PMC4666556

[51] Hamouda, R. et al. Comparative study between zinc oxide nanoparticles synthesis by biogenic and wet chemical methods in vivo and in vitro against *Staphylococcus aureus*. *Microb. Pathog.* **147**, 104384. 10.1016/j.micpath.2020.104384 (2020). 32679246 10.1016/j.micpath.2020.104384

[52] Johansson, D., Rasmussen, M. & Inghammar, M. Thrombocytopenia in bacteraemia and association with bacterial species. *Epidemiol. Infect.* **146**, 1312–1317. 10.1017/s0950268818001206 (2018). 29759089 10.1017/S0950268818001206 PMC9134296

[53] Watson, C. et al. Human platelet activation by *Escherichia coli*: Roles for FcγRIIA and integrin αIIbβ3. *Platelets* **27**, 535–540. 10.3109/09537104.2016.1148129 (2016). 27025455 10.3109/09537104.2016.1148129 PMC5000871

[54] Rigopoulou, E., Gershwin, M. & Bogdanos, D. Immune responses to bacterial infections. In *Liver Immunology Principles and Practice* 3rd edn (eds Gershwin, M. et al.) (Springer, Cham, 2020). 10.1007/978-3-030-51709-0.

[55] Weiner, D., Mitch, W. & Sands, J. Urea and ammonia metabolism and the control of renal nitrogen excretion. *Clin. J. Am. Soc. Nephrol.* **10**, 1444–1458. 10.2215/cjn.10311013 (2015). 25078422 10.2215/CJN.10311013 PMC4527031

[56] Prakash, S. & Chang, T. Microencapsulated genetically engineered live *E.**coli* DHS cells administered orally to maintain normal plasma urea level in uremic rats. *Nat. Med.* **2**, 883. 10.1038/nm0896-883 (1996). 8705857 10.1038/nm0896-883

[57] Al-Taii, D. & Yousif, A. Effects of *E*. *coli* O157:H7 experimental infections on rabbits. *Iraqi J. Vet. Med.* **43**, 34–42. 10.30539/IJVM (2019).

[58] Ezealisiji, K., Noundou, X., Maduelosi, B., Nwachukwu, N. & Krause, R. Green synthesis of zinc oxide nanoparticles using *Solanum torvum* (L) leaf extract and evaluation of the toxicological profile of the ZnO nanoparticles–hydrogel composite in Wistar albino rats. *Int. Nano Lett.* **9**, 99–107. 10.1007/s40089-018-0263-1 (2019).

[59] Siddiqui, S. et al. Biological efficacy of zinc oxide nanoparticles against diabetes: A preliminary study conducted in mice. *Biosci. Rep.* **40**, 20193972. 10.1042/BSR20193972 (2020). 10.1042/BSR20193972 PMC7138905 32207527

[60] Afify, M. et al. Evaluation of zinc-oxide nanoparticles effect on treatment of diabetes in streptozotocin-induced diabetic rats. *Egypt. J. Chem.* **62**, 1771–1783. 10.21608/ejchem.2019.11350.1735 (2019).

[61] Malaikozhundan, B. et al. High synergistic antibacterial, antibiofilm, antidiabetic and antimetabolic activity of *Withania somnifera* leaf extract-assisted zinc oxide nanoparticle. *Bioprocess Biosyst. Eng.* **43**, 1533–1547. 10.1007/s00449-020-02346-0 (2020). 32300871 10.1007/s00449-020-02346-0

[62] Kiyani, M. et al. Antioxidant and anti-gout effects of orally administered zinc oxide nanoparticles in gouty mice. *J. Trace Elem. Med. Biol.* **56**, 169–177. 10.1016/j.jtemb.2019.08.012 (2019). 31479800 10.1016/j.jtemb.2019.08.012

[63] Suganya, K. et al. Anti-gout arthritic activities of ethanolic and zinc oxide nanoparticle extracts of *Citrullus colocynthis*- an in vitro and in silico studies. *Ann. Roman. Soc. Cell Biol.* **25**, 8020–8033 (2021).

[64] Ensign, L. M., Cone, R. & Hanes, J. Oral drug delivery with polymeric nanoparticles: The gastrointestinal mucus barriers. *Adv. Drug Deliv. Rev.* **64**, 557–570. 10.1016/j.addr.2011.12.009 (2012). 22212900 10.1016/j.addr.2011.12.009 PMC3322271

[65] Lee, C. et al. Optical imaging to trace near infrared fluorescent zinc oxide nanoparticles following oral exposure. *Int. J. Nanomed.* **7**, 3203–3209. 10.2147/ijn.s32828 (2012). 10.2147/IJN.S32828 PMC3394462 22811605

[66] Pasupuleti, S. et al. Toxicity of zinc oxide nanoparticles through oral route. *Toxicol. Ind. Health* **28**, 675–686. 10.1177/0748233711420473 (2012). 22033421 10.1177/0748233711420473

[67] Brown, J., Tepperman, B., Hanson, P. & Whittle, B. Lipopolysaccharide induces Ca^2+^-independent nitric oxide synthase activity in rat gastric mucosal cells. *Eur. J. Pharmacol.* **292**, 111–114. 10.1016/0926-6917(94)90033-7 (1994). 7532587 10.1016/0926-6917(94)90033-7

[68] Chong, C. et al. Current updates on the in vivo assessment of zinc oxide nanoparticles toxicity using animal models. *Bio Nano Sci.* **11**, 590–620. 10.1007/s12668-021-00845-2 (2021).

[69] Bohnsack, J. F. & Brown, E. J. The role of the spleen in resistance to infection. *Annu. Rev. Med.* **37**, 49–59. 10.1146/annurev.me.37.020186.000405 (1986). 3518612 10.1146/annurev.me.37.020186.000405

[70] Szabo, G., Romics, L. & Frendl, G. Liver in sepsis and systemic inflammatory response syndrome. *Clin. Liver Dis.* **6**, 1045–1066. 10.1016/s1089-3261(02)00058-2 (2002). 12516206 10.1016/s1089-3261(02)00058-2

[71] Radwan, M., Elzoghby, R., Amin, A., Abo-Sakaya, R. & Hamouda, A. Experimental infection with *E. coli* O157 in rats and its toxic effect, biochemical and histopathological changes with referee to modern therapy. *Ann. Microbiol. Immunol.* **4**, 1–10 (2021).

[72] Abd El Megid, A., Khaled, M., Emam, M. & Adel, A. Biochemical role of zinc oxide and propolis nanoparticles in protection rabbits against coccidiosis. *Benha Vet. Med. J.* **34**, 314–328. 10.21608/bvmj.2018.54256 (2018).

[73] Sohail, M. et al. Green synthesis of zinc oxide nanoparticles by Neem extract as multi-facet therapeutic agents. *J. Drug Deliv. Sci. Technol.* **59**, 101911. 10.1016/j.jddst.2020.101911 (2020).

[74] Hatab, M. H., Rashad, E., Saleh, H. M., El-Sayed, E. R. & AbuTaleb, A. M. Effects of dietary supplementation of myco-fabricated zinc oxide nanoparticles on performance, histological changes, and tissues Zn concentration in broiler chicks. *Sci. Rep.* **12**, 18791. 10.1038/s41598-022-22836-3 (2022). 36335156 10.1038/s41598-022-22836-3 PMC9637221

[75] Plotnikov, E. et al. Mechanisms of LPS-induced acute kidney injury in neonatal and adult rats. *Antioxidants* **7**, 105. 10.3390/antiox7080105 (2018). 30096767 10.3390/antiox7080105 PMC6115895

[76] Wang, C. et al. Alpha-hemolysin of uropathogenic *Escherichia coli* induces GM-CSF-mediated acute kidney injury. *Mucosal Immunol.* **13**, 22–33. 10.1038/s41385-019-0225-6 (2020). 31719643 10.1038/s41385-019-0225-6 PMC6914670

[77] Tufft, L. S. & Nockels, C. F. The effects of stress, *Escherichia coli,* dietary ethylenediaminetetraacetic acid, and their interaction on tissue trace elements in chicks. *Poul. Sci.* **70**, 2439–2449. 10.3382/ps.0702439 (1991). 10.3382/ps.0702439 1784566

[78] Shah, A. et al. Histopathological and hematological investigations of mice model inoculated with nickel oxide nanoparticles and bacterial pathogens: In-vitro and in-vivo antibacterial studies. *J. King Saud Univ. Sci.* **35**, 102456. 10.1016/j.jksus.2022.102456 (2023).

[79] Singh, N., Das, M., Gautam, R., Ramteke, A. & Rajamani, P. Assessment of intermittent exposure of zinc oxide nanoparticle (ZNP)–mediated toxicity and biochemical alterations in the splenocytes of male Wistar rat. *Environ. Sci. Pollut. Res. Int.* **26**, 33642–33653. 10.1007/s11356-019-06225-4 (2019). 31588521 10.1007/s11356-019-06225-4

[80] Sebai, H., Sani, M., Aouani, E. & Boughanmi, N. G. Cardioprotective effect of resveratrol on lipopolysaccharide-induced oxidative stress in rat. *Drug Chem. Toxicol.* **34**, 146–150. 10.3109/01480545.2010.494666 (2011). 21314464 10.3109/01480545.2010.494666

[81] Rezaei, S., Naghadeh, B., Nazarizadeh, A. & Sabzikar, Z. Comparative study of cardio-protective effects of zinc oxide nanoparticles and zinc sulfate in streptozotocin-induced diabetic rats. *J. Trace Elem. Med. Biol.* **42**, 129–141. 10.1016/j.jtemb.2017.04.013 (2017). 28595785 10.1016/j.jtemb.2017.04.013

[82] Saman, S., Moradhaseli, S., Shokouhian, A. & Ghorbani, M. Histopathological effects of ZnO nanoparticles on liver and heart tissues in Wistar rats. *Adv. Biores.* **4**, 83–88 (2013).

[83] Abdel-Magied, N. & Shedid, S. Impact of zinc oxide nanoparticles on thioredoxin-interacting protein and asymmetric dimethylarginine as biochemical indicators of cardiovascular disorders in gamma-irradiated rats. *Environ. Toxicol.* **35**, 430–442. 10.1002/tox.22879 (2020). 31749214 10.1002/tox.22879

[84] Adamu, S., Umaru, H., Albert, H. & Muhammad, A. The effect of green synthesized zinc oxide nanoparticles using *Allium cepa* extracts on Triton X-100 induced hyperlipidemia in rats. *Int. J. Nut. Sci.* **8**, 36–46. 10.30476/ijns.2023.97566.1214 (2023).

[85] Clinical and Laboratory Standards Institute CLSI. M100: Performance standards for antimicrobial susceptibility testing. 30^th^ Edition, **40** (2020).

[86] Zimbro, M. J., Power, D. A., Miller, S. M., Wilson, G. E. & Johnson, J. A. *Difco & BBL Manual of Microbiological Culture Media* 2nd edn. (Becton Dickinson and Company, Maryland, USA, 2009).

[87] White, T. J., Bruns, T., Lee, S. & Taylor, J. Amplification and direct sequencing of fungal ribosomal RNA genes for phylogenetics. In *PCR Protocols: A Guide to Methods and Applications* (eds Innis, M. A. et al.) (Academic Press, San Diego, U.S.A, 1990).

[88] Clinical and Laboratory Standards Institute CLSI. M07-A10: Methods for dilution antimicrobial susceptibility tests for bacteria that grow aerobically; approved standard. 10^th^ Edn. **35** (2015).

[89] Snedecor, G. W. & Cochran, W. G. *Statistical methods* 7th edn. (Oxford and J.B.H. Publishing, Amesterdam, 1980).

